# Activation and detoxification of cassava cyanogenic glucosides by the whitefly *Bemisia tabaci*

**DOI:** 10.1038/s41598-021-92553-w

**Published:** 2021-06-24

**Authors:** Michael L. A. E. Easson, Osnat Malka, Christian Paetz, Anna Hojná, Michael Reichelt, Beate Stein, Sharon van Brunschot, Ester Feldmesser, Lahcen Campbell, John Colvin, Stephan Winter, Shai Morin, Jonathan Gershenzon, Daniel G. Vassão

**Affiliations:** 1 Max Planck Institute for Chemical Ecology, 07745 Jena, Germany; 2 The Hebrew University of Jerusalem, 7610001 Rehovot, Israel; 3 Leibniz Institute DSMZ-German Collection of Microorganisms and Cell Cultures, 38104 Braunschweig, Germany; 4 Natural Resources Institute, University of Greenwich, Chatham Maritime, ME4 4TB Kent UK; 5 University of Queensland, Brisbane, QLD 4072 Australia; 6 Weizmann Institute of Science, 7610001 Rehovot, Israel; 7 EMBL-European Bioinformatics Institute, Cambridge, CB10 1SD UK

**Keywords:** Entomology, Plant ecology, Secondary metabolism, Chemical ecology, Natural products

## Abstract

Two-component plant defenses such as cyanogenic glucosides are produced by many plant species, but phloem-feeding herbivores have long been thought not to activate these defenses due to their mode of feeding, which causes only minimal tissue damage. Here, however, we report that cyanogenic glycoside defenses from cassava (*Manihot esculenta*), a major staple crop in Africa, are activated during feeding by a pest insect, the whitefly *Bemisia tabaci*, and the resulting hydrogen cyanide is detoxified by conversion to beta-cyanoalanine. Additionally, *B. tabaci* was found to utilize two metabolic mechanisms to detoxify cyanogenic glucosides by conversion to non-activatable derivatives. First, the cyanogenic glycoside linamarin was glucosylated 1–4 times in succession in a reaction catalyzed by two *B. tabaci* glycoside hydrolase family 13 enzymes in vitro utilizing sucrose as a co-substrate. Second, both linamarin and the glucosylated linamarin derivatives were phosphorylated. Both phosphorylation and glucosidation of linamarin render this plant pro-toxin inert to the activating plant enzyme linamarase, and thus these metabolic transformations can be considered pre-emptive detoxification strategies to avoid cyanogenesis.

## Introduction

Many plants produce two-component chemical defenses as protection against attacks from herbivores and pathogens. In these plants, protoxins that are often chemically protected by a glucose residue are activated by an enzyme such as a glycoside hydrolase yielding an unstable aglycone that is toxic or rearranges to form toxic products^1^. The glycoside and the hydrolase are stored in separate compartments that mix upon plant damage, activating the toxin when the plant is under attack. Two-component defenses include cyanogenic, benzoxazinoid and iridoid glycosides and glucosinolates, and have long been known to play decisive roles in interactions between plants and herbivores, especially when extensive plant tissue disruption happens during feeding, such as during attack by chewing herbivores^1,2^. However, the activation of such defenses by piercing-sucking, phloem-feeding herbivores such as aphids and whiteflies is poorly understood^3^ in spite of the agricultural importance of these insects.

Cyanogenic glycosides are widespread two-component chemical defenses in plants that release the respiratory toxin hydrogen cyanide upon activation^4–6^. Cyanogenic glycosides are *O-*β-glycosides of α-hydroxynitriles, which are classified as aliphatic or aromatic, depending on the amino acid from which they are derived^7^. They also occur as disaccharides in some plant species, which may serve as stable transport forms^8,9^. Crop plants that produce cyanogenic glycosides include several legumes and fruits, as well as the tropical root crop cassava (manioc, yuca; *Manihot esculenta*). Cassava originated in the Amazon basin^10^ and was introduced to Africa in the sixteenth century^10^, where it has become an extremely important source of carbohydrates, especially for smallholder farmers^11,12^ with production expected to grow to 100 Mt in Sub-Saharan Africa by 2025^11^. The main cyanogenic glycoside present in cassava is a valine-derived cyanogenic mono-glycoside called linamarin, highly abundant in both aerial and root tissues of this plant^13^, reaching a level that can generate up to 2 mg cyanide per g dry weight^14^. The hydrolytic enzyme required for the activation of linamarin and other cyanogenic glycosides in cassava is a β-glucosidase commonly referred to as linamarase^13,15^. Upon tissue damage, this enzyme cleaves linamarin to give an unstable hydroxynitrile that rearranges to produce hydrogen cyanide and acetone. Rearrangement of the hydroxynitrile occurs spontaneously but can be accelerated by an enzyme known as hydroxynitrile lyase^16^. As a crop, cassava displays resistance to drought, but is challenged by several viral diseases (e.g. cassava mosaic, cassava brown streak, and cassava vein mosaic viruses)^17^, many of which are vectored by the whitefly *Bemisia tabaci* when feeding on cassava phloem tissue^18^. Viral epidemics have become more severe in Africa in recent years due to large outbreaks of whiteflies^12^.

The whitefly *B. tabaci* is a complex of cryptic species that are morphologically indistinguishable^19^. Collectively they are polyphagous phloem feeders able to feed on over 600 species of plants^20^, and are an important world-wide crop pest in part because of their ability to vector over 300 plant viruses^21^. As a consequence, discovery of the mechanisms by which species in the *B. tabaci* complex feed so successfully on crops could lead to new control measures for *B. tabaci* on cassava and other crops^12,21,22^.

For whiteflies and other phloem-feeding herbivores, two-component defenses are being increasingly implicated in their interactions with host plants^23–29^. Glucosinolates have been shown to affect aphid feeding, with certain indolic glucosinolate hydrolysis products and downstream metabolites being detected after *Myzus persicae* ingestion^27^. Glucosinolates were also observed to alter *B. tabaci* performance and host selection^29^, and this insect has been shown to detoxify glucosinolates via the formation of desulpho-glucosinolates^30^, mercapturic acid pathway metabolites and the transfer of additional glucose residues^28^. Moreover, the activities of cyanide detoxification enzymes in *B. tabaci* are increased when feeding on cassava in comparison to sweet potato (a non-cyanogenic plant)^23^.

Here we provide evidence concerning the interaction of cassava cyanogenic glycosides with the phloem feeder *B. tabaci*. We first report the activation of cyanogenic glycosides upon feeding as seen by the formation of the hydrogen cyanide detoxification product beta-cyanoalanine^31^. Then we describe the detoxification of cyanogenic glycosides via both transglucosidation and phosphorylation of their sugar moieties. Since the glucosylated and phosphorylated derivatives are resistant to hydrolysis by the plant activating enzyme linamarase, they therefore represent non-toxic derivatives.

## Results

### Cassava cyanogenic glycosides are activated by *B. tabaci* feeding

**Figure 1:**
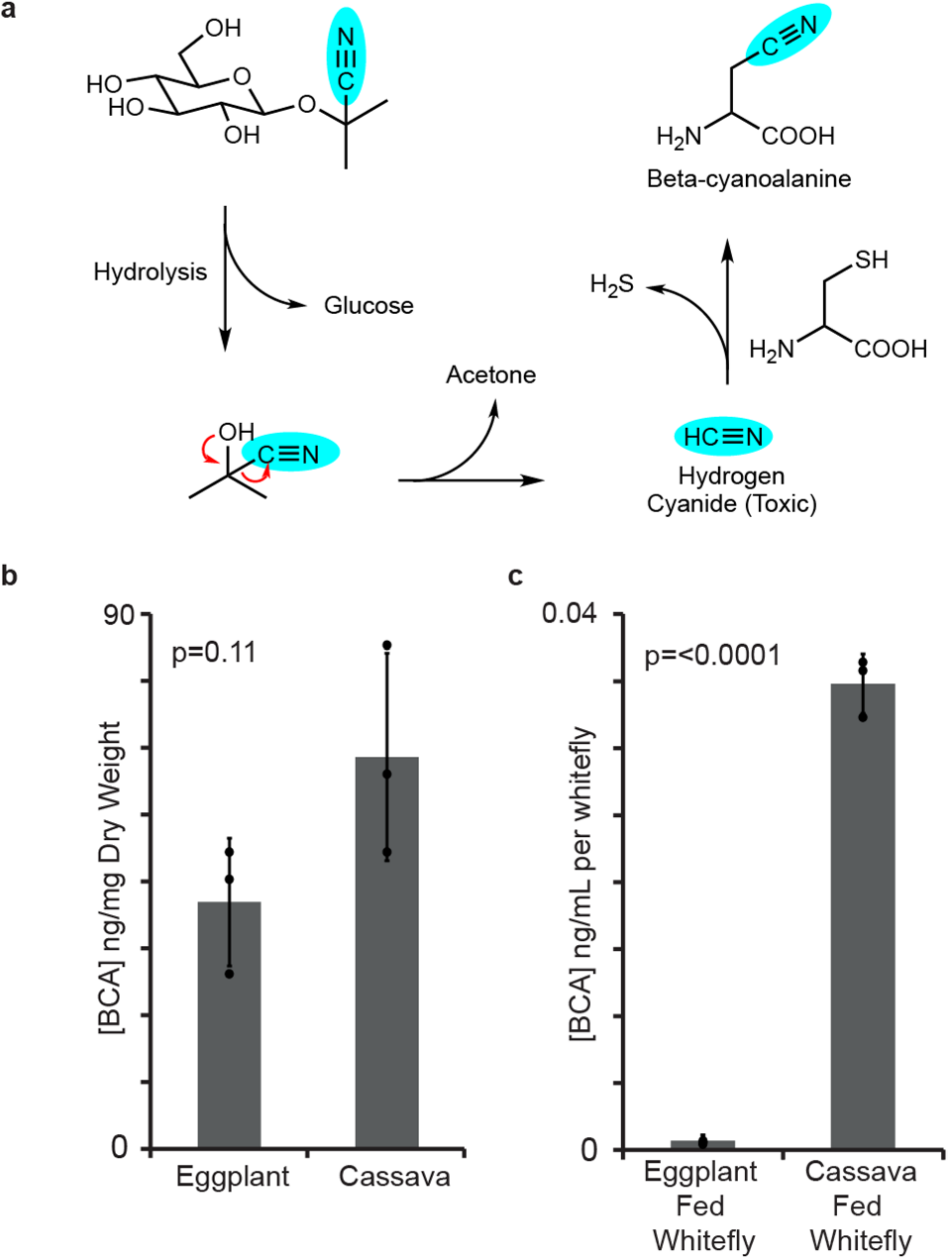
Beta-cyanoalanine levels support the activation of cassava cyanogenic glycosides during *B. tabaci* feeding. (**a**) Scheme showing the hydrolysis of the cyanogenic glycoside linamarin and the detoxification of the hydrogen cyanide released via beta-cyanoalanine formation. (**b**) Endogenous beta-cyanoalanine (BCA) concentrations in both eggplant and cassava leaves were not different (amounts depicted have been normalized per plant dry weight, P-value from unpaired t-test with N = 3). (**c**) Extracts from groups of 200 *B. tabaci* SSA1-SG3 adults feeding on cassava contained elevated levels of BCA in comparison to extracts from eggplant-fed insects (amounts depicted have been normalized per insect, P-value from unpaired t-test with N = 3).

The hydrolysis of cyanogenic glycosides during whitefly feeding leads to the release of the notorious respiratory toxin hydrogen cyanide. To assess the extent of this activation, we investigated the levels of a well-known cyanide detoxification product, beta-cyanoalanine (Fig. 1a), in *B. tabaci* species SSA1-SG3 (BtSSA1-SG3), a species with an extended host range that feeds on cassava^32^. BtSSA1-SG3 adults feeding on leaves of the cyanogenic plant cassava (*M. esculenta*) were compared to those feeding on a non-cyanogenic plant, eggplant (*Solanum melongena*). Since all plants produce beta-cyanoalanine as a natural by-product of ethylene biosynthesis^31,33^ the concentrations of this amino acid were also measured in the tissues of both of these plant species to determine the endogenous levels of this compound in fresh plants . The levels of beta-cyanoalanine in cassava and eggplant were not different from each other (p = 0.11, N = 3) (Fig. 1b); however, BtSSA1-SG3 adults produced much higher levels of this compound while feeding on cassava than while feeding on eggplant (p < 0.0001, N = 3) (Fig. 1c). The accumulation of beta-cyanoalanine in the BtSSA1-SG3 whiteflies during feeding on cassava provides strong evidence for the activation of cyanogenic glycosides.

### Cassava cyanogenic glycosides are glucosylated by *B. tabaci*

Honeydew from BtSSA1-SG3 adults feeding on cassava was collected and chemically analyzed via an untargeted LC–MS approach for the presence of the cyanogenic glycoside linamarin and possible derivatives. A peak corresponding to the native glycoside was easily observed, confirming that this insect was exposed to linamarin while feeding on cassava phloem sap. Additionally, MS signals consistent with glycosylated linamarin derivatives were also detected. The molecular masses of these putative products presented a characteristic serial mass addition of + 162 Da units up to + 648 Da, suggestive of glucosylation, with elution times being slightly shorter with each addition. These peaks were also detected in honeydew from the *B. tabaci* species MEAM1 (BtMEAM1, a broad generalist not naturally found feeding on cassava)^32^ when these whiteflies were fed artificial diets consisting of sucrose and linamarin, but not when fed sucrose alone, confirming they were linamarin derivatives produced by the insects.

**Figure 2:**
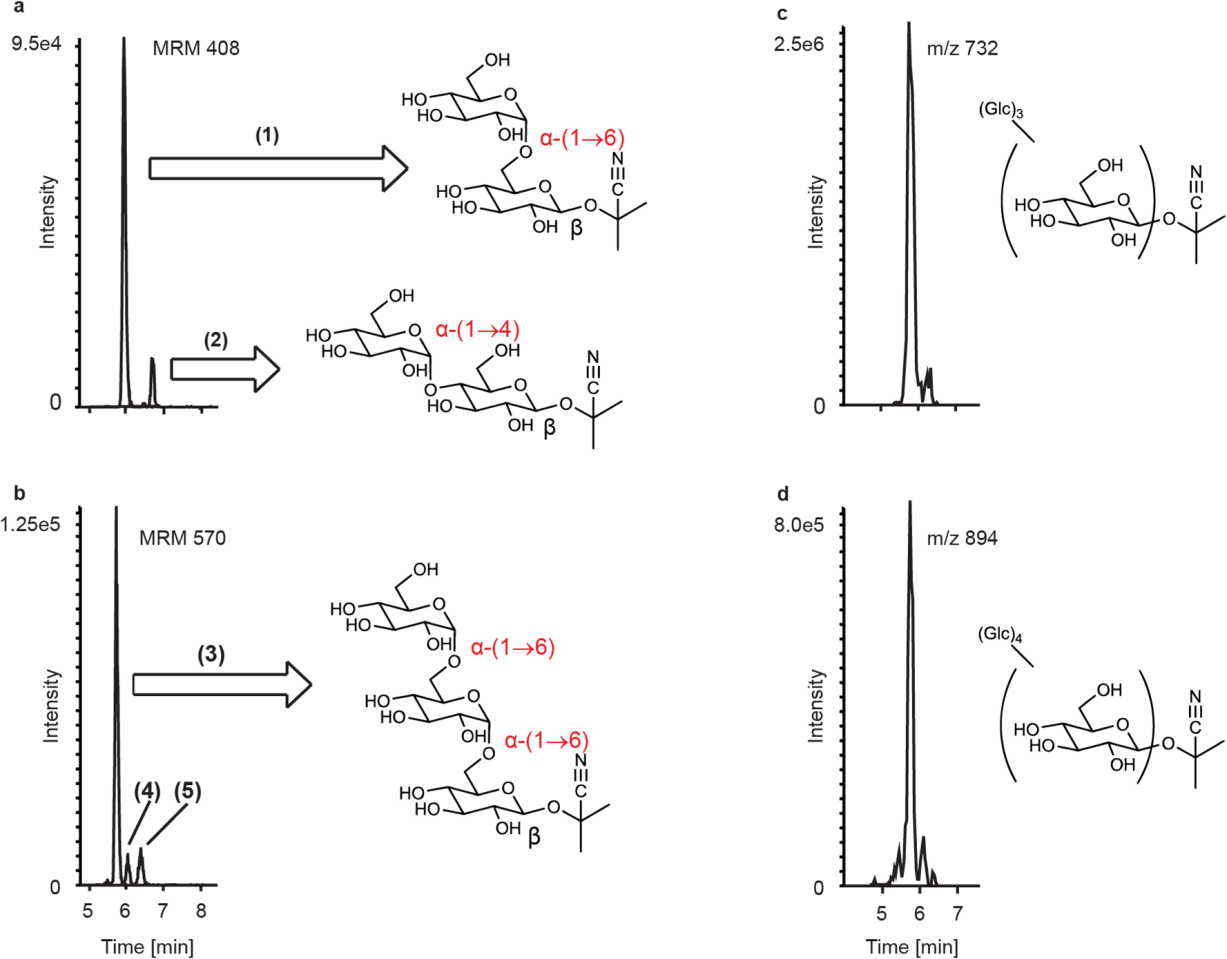
Novel linamarin-derived metabolites identified in *B. tabaci* honeydew. Linamarin-derived metabolites in the honeydew contain additional alpha-linked glucose moieties. (**a**) shows metabolites corresponding to 1 glucose addition (compounds (**1**) and (**2**)) and (**b**) shows metabolite peaks corresponding to 2 glucose additions (compounds (**3**), (**4**) and (**5**)). Metabolites with up to 4 further hexose additions were detected via untargeted analysis (**c** and **d**). Compounds (**1**), (**2**) and (**3**) were purified from honeydew and their structures determined by MS and NMR (Supplementary Note 1).

In order to elucidate the structure of these whitefly metabolites of linamarin, the compounds resulting from the addition of one and two apparent glucose moieties, which corresponded to the most intense MS signals in the series of glucosylated derivatives, were purified using HPLC fractionation. NMR analyses revealed that the structures of these compounds were indeed glucosylated linamarin conjugates. Sugar addition to the original glucose of linamarin occurred in either an α-(1→6) or α-(1→4) orientation (compounds **1** and **2**, respectively) with the latter having slightly greater retention. The diglucose derivative showed the serial addition of two α-(1→6) linked glucose moieties to the previously existing β-linked glucose (compound **3**) (Fig. 2a,b and NMR Supplementary Note). Quantitative ^1^H-NMR using sucrose as an external standard was utilized to estimate the amount of the monoglucose derivative **1** and diglucose derivative **3** purified (5.88 µg and 3.38 µg respectively). LC–MS standard curves for these purified metabolites were then constructed, allowing for the calculation of molar ratios for these compounds in comparison to intact linamarin in the honeydew of BtSSA1-SG3 adults feeding on cassava. It was found that glycosylated linamarin derivatives corresponding to 1 and 2 glucose additions were present in the honeydew in a combined 5.62:1 (SE = 0.89, N = 3) ratio to intact linamarin. Additional peaks with mass spectra consistent with two glucose additions were also observed (Compounds **4** and **5**); however the quantities and purities of the fractions obtained after chromatographic separation were not sufficient for adequate structure elucidation. Glycosides with masses corresponding to three and four glucose additions to the original β-linked glucose were also detected (Fig. 2c,d).

### Cassava cyanogenic glycosides are also phosphorylated by *B. tabaci*

**Figure 3:**
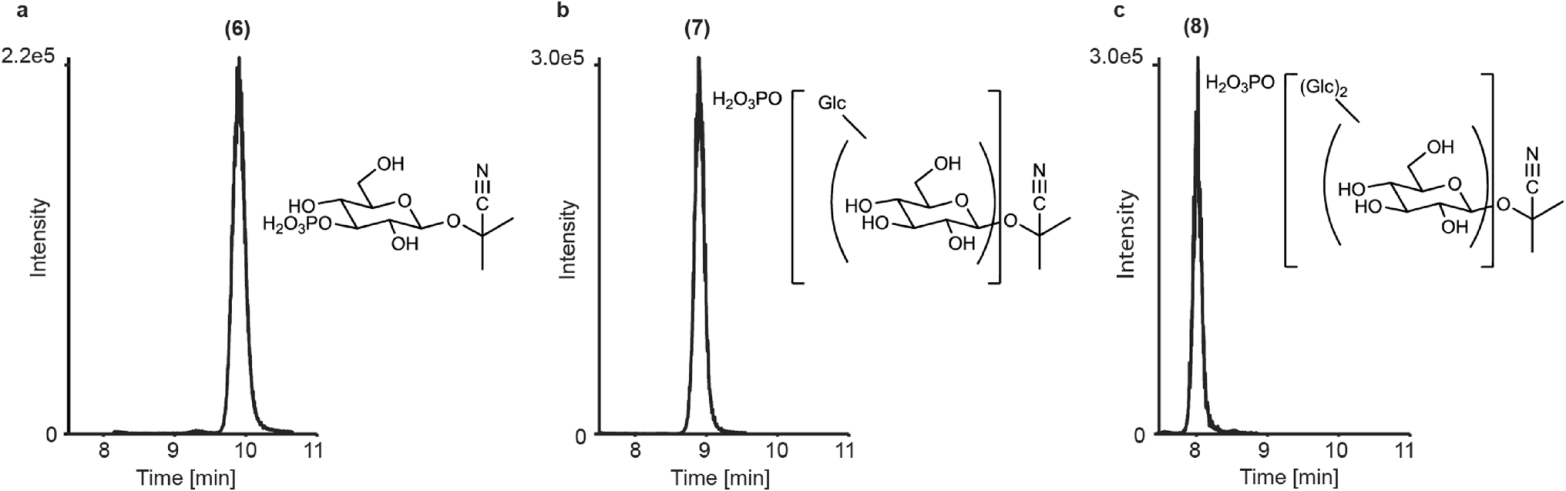
Novel phosphorylated linamarin derivatives in *B. tabaci* honeydew. Linamarin and the whitefly-produced linamarin glycosides were also phosphorylated by *B. tabaci*. (**a**) The position for phosphorylation of linamarin was elucidated as C3 (**6**) by NMR (Supplementary Note 1). The structures of both the monoglucosylated (**7**, **b**) and diglucosylated (**8**, **c**) phosphorylated derivatives were supported by mass spectral data, but NMR analysis was not possible due to low abundance.

In addition to glucosides, additional unknown metabolites were detected in the honeydew of BtSSA1-SG3 whiteflies that fed on cassava and BtMEAM1 whiteflies that fed on linamarin-containing artificial diets, but these metabolites were absent in diets not containing linamarin. These unknown compounds again showed an MS pattern suggesting serial glucose additions (+ 162_n_), with progressively earlier eluting peaks. The smallest of these metabolites displayed a mass of 326 Da (Fig. 3a) (**6**), which is 80 mass units greater than linamarin, but eluting much later. Two earlier eluting metabolites consistent with + 162 and + 324 Da additions to **6** (Fig. 3b,c, compounds **7** and **8** respectively) were also observed. We hypothesized that the addition of 80 Da could correspond to either a sulphate or phosphate group linked to linamarin, with the earlier eluting peaks being a result of subsequent glycosylation. The addition of a phosphate was supported by the accurate mass data (Supplemental Figure S1 and Supplementary Table S1) and the disappearance of these metabolites upon incubation with alkaline phosphatase (Supplemental Figure S2). Purification via HPLC fractionation followed by NMR analysis revealed the addition of a phosphate moiety bound to linamarin at position 3 of the β-linked sugar (**6**) based on the deshielding of the ^1^H and ^13^C signals at this position (NMR Supplementary Note). Due to the low abundance of the purified products, no NMR spectroscopic evidence could be obtained for the glucosylated phosphate derivatives.

### Linamarin metabolites produced by *B. tabaci* resist enzymatic activation

**Figure 4:**
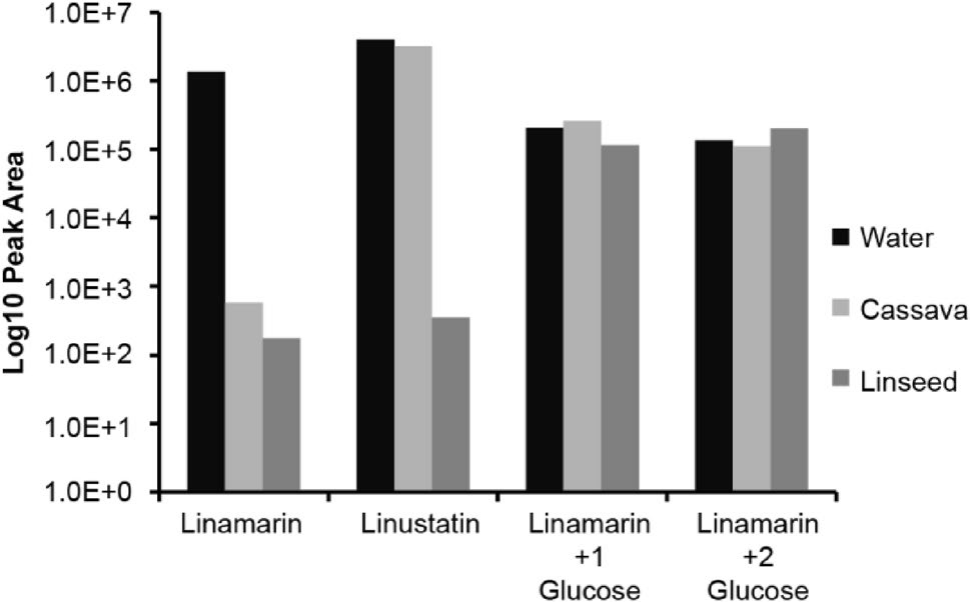
Resistance of glucosylated linamarin derivatives to activation by plant enzymes with release of hydrogen cyanide. The cyanogenic monoglucoside linamarin, the cyanogenic diglucoside linustatin and the glucosylated linamarin derivatives found in the honeydew of whiteflies fed on cassava were tested. Honeydew and standards were incubated with crude enzyme extracts from cassava and linseed or with water alone. Linamarin was readily hydrolyzed by both cassava and linseed extracts, and linustatin was hydrolyzed by linseed enzymes. Conversely, the insect-derived glycosides were not substrates for either of the enzyme extracts.

In order to determine whether the linamarin glycosides and phosphorylated metabolites can be activated by plant enzymes to form hydrogen cyanide similarly to linamarin, extracts of cassava leaves containing linamarase activity, as well as linseed extracts containing linustatinase^8,34^ (a disaccharidase) activity were incubated with pure linamarin and with the honeydew of cassava-fed *B. tabaci*. Linamarin was degraded in the presence of both cassava enzyme extracts and linseed enzyme extracts, while the disaccharide linustatin was only hydrolyzed in the presence of the linseed enzyme extract. The insect-derived glycosides, however, remained unhydrolyzed in the presence of both enzyme extracts, resisting both plant monosaccharidase and disaccharidase activities (Fig. 4). Phosphorylated linamarin and phosphorylated linamarin glycosides were also stable to enzymatic activation in the presence of the cassava extracts (Supplemental Figure S3). Therefore, formation of these derivatives likely serves as a true pre-emptive detoxification of linamarin rendering products that can no longer be hydrolyzed with the release of hydrogen cyanide.

### Mechanism of glucose addition to the cyanogenic glycoside linamarin

**Figure 5:**
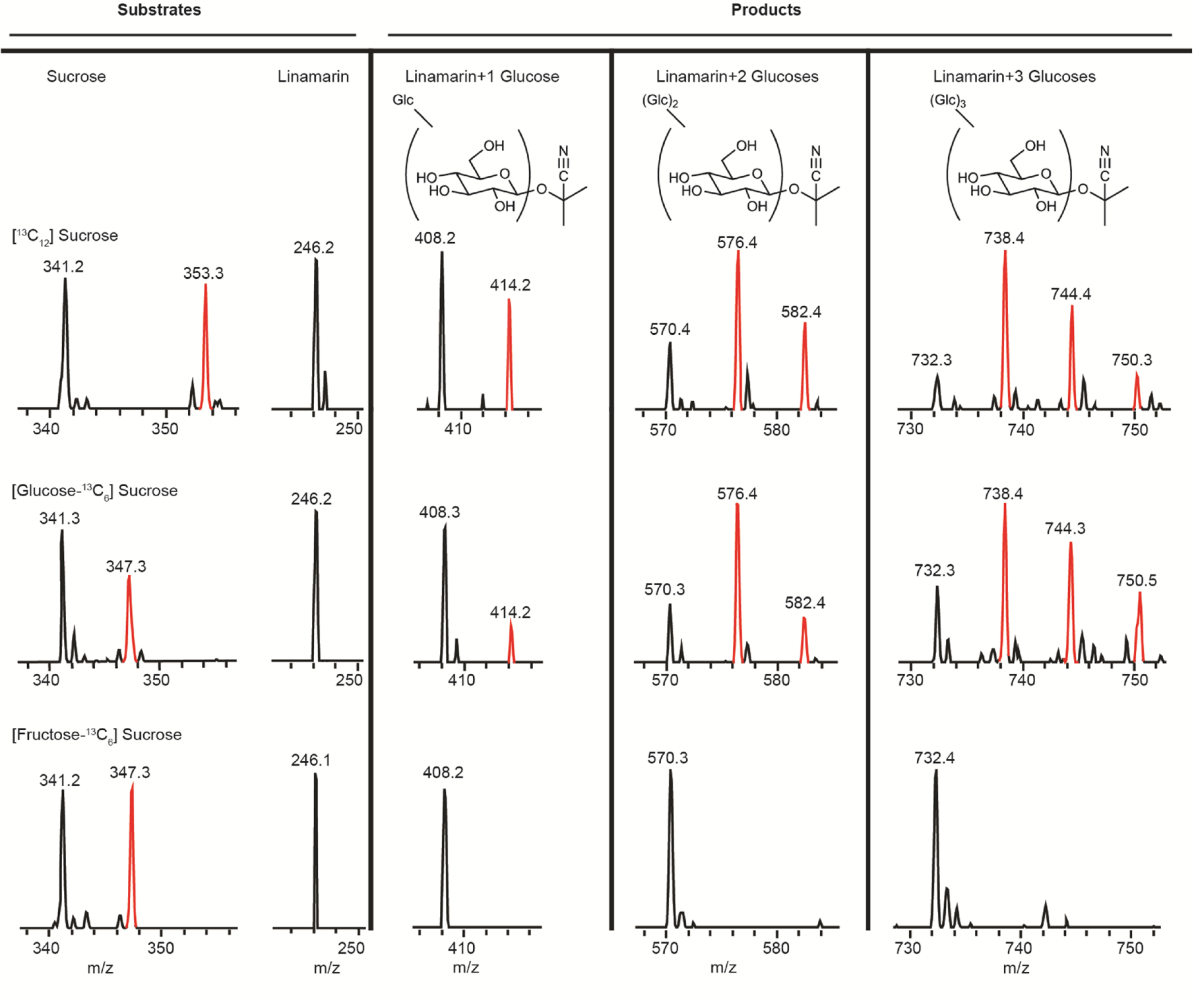
Evidence for enzymatic mechanism of linamarin glucosylation by *B. tabaci*. Glucosylation of cyanogenic glycosides in *B. tabaci* is catalyzed by a transglucosidase activity based on feeding of [^13^C] sucrose isotopologues and linamarin to insects in artificial diet. Shown are MS regions from LC–MS analyses of substrates and products of feeding experiments on three different diets. Feeding fully ^13^C-labeled sucrose and sucrose ^13^C-labeled in the glucose moiety gave labeling in the newly added glucose of glucosylated linamarin metabolites. However, feeding sucrose ^13^C-labeled in the fructose moiety gave no label in the products, demonstrating incorporation of only the glucose of sucrose into the products. Feeding of free ^13^C-labeled glucose and fructose with linamarin also resulted in no incorporation into glucosylated derivatives of linamarin (Supplementary Figure S4), showing that the glucose must originate from sucrose as expected in transglucosidase catalysis.

Glucosylation reactions are most often catalyzed by either of two enzyme classes, UDP-glucosyl-transferases and transglucosidases. The latter of these two classes utilizes a mechanism which transfers a glucose unit from a donor disaccharide directly to an acceptor molecule, while the former utilizes an activated form of glucose (UDP-glucose). In order to elucidate the mechanism by which glucose units are added to linamarin by whiteflies, experiments were carried out to feed ^13^C sucrose isotopologues in artificial diet. Labeled glucose was incorporated into the glycosides of linamarin when diets contained [^13^C_12_]sucrose and [glucose-^13^C_6_]sucrose, but not when diets contained [fructose-^13^C_6_]sucrose, [^13^C_6_]glucose or [^13^C_6_]fructose (Fig. 5 and Supplemental Figure S4). Label was also incorporated into phosphorylated glycosides in the same manner (Supplemental Figure S5). This mechanism is consistent with a transglucosidase activity, which is typically carried out by enzymes of the glycoside hydrolase (GH) family^35^.

### Expression profiles of the transglucosidase genes *BtSUC2* and *BtSUC5* in *B. tabaci* adults feeding on various host plants

**Figure 6:**
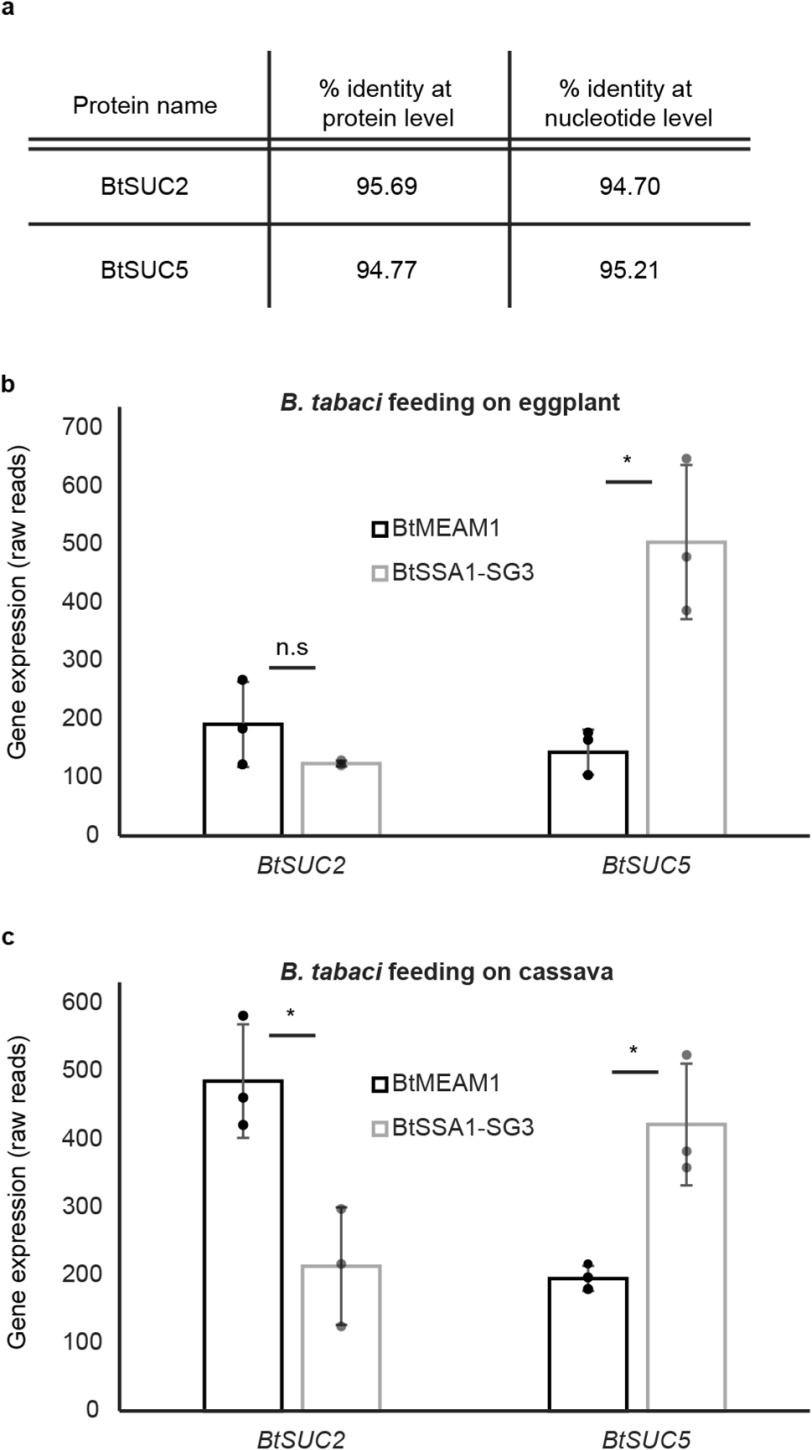
Comparison of transglucosidase BtSUC2 and BtSUC5 sequences in *B. tabaci* species and relative gene expression in different species on different food plants. (**a**) The percent sequence identities of SSA1-SG3 homologues of *BtSUC2* and *BtSUC5* to the originally isolated genes from BtMEAM1 were determined at the DNA and protein levels. (**b**,**c**) Mean numbers of reads mapped to *BtSUC2* and *BtSUC5* were plotted in the species BtMEAM1 and BtSSA1-SG3 feeding on two host plants, eggplant and cassava. Asterisks denote statistically significant differences (p value < 0.05) between means of biological replicates (N = 3) with individual data overlaid as points on the histogram.

BtSUC2 and BtSUC5 were previously identified in the whitefly species BtMEAM1 via phylogenetic analyses followed by heterologous production and biochemical characterization as transglucosidases that glucosylate glucosinolates, thereby preventing their hydrolysis and release of toxic products^28^. Thus we investigated their potential to participate in the glucosylation of cassava cyanogenic glycosides using BtSSA1-SG3 that feeds on cassava and BtMEAM1, which is not naturally found on cassava. First the genomic sequences of *BtSUC2* and *BtSUC5* in the cassava-feeding BtSSA1-SG3 were found to be approximately 95% identical to their counterparts in BtMEAM1 (Fig. 6a). Next, the expression of the *BtSUC2* and *BtSUC5* genes in terms of reads mapping to their exons was compared in the two whitefly species feeding on two different plant species (using raw RNA-Seq data from SRP127757^32,36^). When feeding on eggplant (no cyanogenic glycosides), *BtSUC2* expression did not differ between the species (p = 0.187, N = 3), but *BtSUC5* expression was significantly higher in BtSSA1-SG3 than in BtMEAM1 (p = 0.011, N = 3) (Fig. 6b). When feeding on cassava (containing cyanogenic glycosides), *BtSUC2* expression was significantly higher in BtMEAM1 than in BtSSA1-SG3 (p = 0.017, N = 3), but the pattern for *BtSUC5* was almost identical to that on eggplant, with expression in BtSSA1-SG3 significantly higher than in BtMEAM1 (p = 0.013, N = 3) (Fig. 6c).

### In vitro activities of the transglucosidases BtSUC2 and BtSUC5

**Figure 7:**
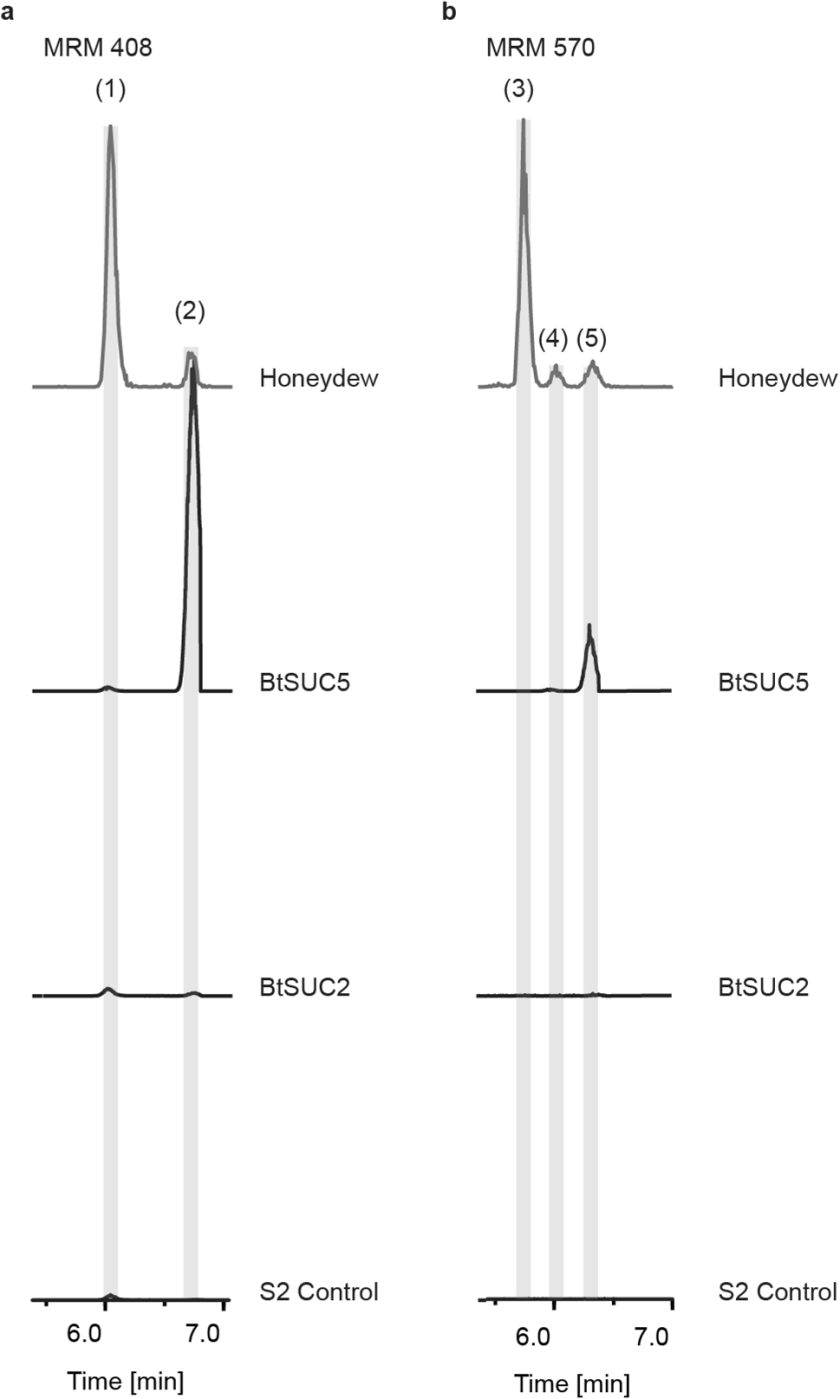
Chromatographic analyses of glucosylated products catalyzed by the *B. tabaci* transglucosidases BtSUC2 and 5. Medium of *Drosophila* S2 cells expressing these enzymes was assayed with linamarin and sucrose. Depicted are extracted multiple reaction monitoring (MRM) LC–MS chromatograms for mono- and di-glucosylated linamarin derivatives. (**a**) BtSUC2 and 5 showed transglucosidation activity, producing α-(1→4)-linked glucose derivatives (**2**) of linamarin with BtSUC2 also producing small amounts of the α-(1→6)-linked derivative (**1**) above control levels. (**b**) BtSUC5 additionally produced the diglycosylated product (**5**). The S2 control assay was performed on medium extracts of untransformed S2 cells. (**1**: α-(1→6)-linked glucose derivative of linamarin; **2**: α-(1→4)-linked glucose derivative of linamarin; **3**: α-(1→6), α-(1→6)-linked diglucosylated derivative of linamarin; **4**, **5**: unknown diglucosylated derivatives of linamarin).

*Drosophila* S2 cells producing BtSUC2 and BtSUC5 were utilized in enzyme assays with the cyanogenic glycoside linamarin and the donor disaccharide sucrose. BtSUC2 produced the α-(1→4) glycoside of linamarin (**2**) approximately 50 times more efficiently than S2 control cells, and smaller amounts of the α-(1→6) derivative **1** (approximately 8 times more efficiently than control cells) (Fig. 7a). No higher order glycosides were formed by BtSUC2 (Fig. 7b). BtSUC5 on the other hand produced much larger amounts of **2** (more than 50,000 times more efficient than S2 control cells) (Fig. 7a), as well as a glycoside corresponding to a further glucose addition (**5**) (Fig. 7b) with a similar efficiency. BtSUC5 also produced a smaller amount of **1** (approximately 7 times more active than controls) and a peak corresponding to an unknown derivative (**4**) (Fig. 7b). BtSUC5 displayed apparent Michaelis–Menten kinetics, with an estimated *K*_M_ of ~ 0.5 mM for linamarin and ~ 0.13 M for sucrose (Supplemental Figure S6). For BtSUC2, however, the low activity towards linamarin prevented an estimation of *K*_M_ values. BtSUC2 and BtSUC5 activities towards the purified phosphorylated linamarin derivatives **6** and **7** were also investigated, and both enzymes glucosylated **6** forming a product with very slightly different retention time compared to **7** (Supplemental Figure S7a), suggesting a positional isomer. While BtSUC2 did not use **7** as substrate, BtSUC5 produced a compound corresponding by mass to **8**, but with a different retention time (Supplemental Figure S7b). The precise amounts of the phosphorylated derivatives used in these assays as substrates were not determined due to their low abundance.

BtSUC2 and BtSUC5 were also assayed with other plant-derived glucosides as potential substrates. Specialized plant metabolites from various two-component defensive compound classes were tested including benzoxazinoid, cyanogenic, phenolic, iridoid, flavonoid and other cyanogenic glycosides, as well as glucosinolates. BtSUC2 and 5 had detectable transglucosidase activity with all substrates (Supplemental Figure S8), with BtSUC5 having the greatest activity of the two proteins and the flavonoid rutin having the least activity of the nine substrates.

## Discussion

Phloem-feeding insects were once thought to be largely unaffected by two-component plant defenses such as cyanogenic glycosides and glucosinolates, as their feeding mode does not appear to cause sufficient tissue damage to initiate hydrolytic activation of these compounds^37^. However, increasing evidence suggests that phloem feeding can indeed cause such activation^23,24,26,29^. We recently demonstrated the activation of aliphatic glucosinolates by *B. tabaci* feeding on plants of the Brassicaceae after finding isothiocyanate hydrolysis products and their detoxified glutathione conjugates in the honeydew^28^. Here, we report that *B. tabaci* feeding on the cyanogenic glycoside-producing plant cassava accumulates beta-cyanoalanine, a cysteine conjugate of hydrogen cyanide that is considered to be a detoxification product^38^, in their bodies. Whiteflies fed on cassava contained 50 times greater amounts of beta-cyanoalanine than their counterparts fed on a non-cyanogenic plant, eggplant (Fig. 1). The residual beta-cyanoalanine found after eggplant feeding is likely of plant and not insect origin since this amino acid is also produced by plants upon detoxification of the cyanide formed during the biosynthesis of the hormone ethylene^31,33^. Undamaged cassava and eggplant tissues contained similar amounts of beta-cyanoalanine. Therefore the drastically higher levels of beta-cyanoalanine in *B. tabaci* when feeding on cassava instead of eggplant suggest an enzymatic conversion of liberated cyanide from the cyanogenic glycosides of cassava. While beta-cyanoalanine synthase activity has been reported in whitefly enzyme extracts via the production of H_2_S^23^, no specific *B. tabaci* enzymes have been identified that could catalyze this transformation. Activation of cassava cyanogenic glycosides would be presumed to happen during whitefly feeding, upon stylet penetration or navigation to the phloem, or in the gut of the whitefly post ingestion.

Since cyanogenic glycosides are activated by hydrolysis upon whitefly feeding, we explored whether this insect possessed the ability to metabolize the chief cassava cyanogenic glycoside linamarin to a non-hydrolyzable product prior to its potential activation. We found glucosylated linamarin derivatives in the honeydew of whiteflies feeding on cassava with the additions of α-glucosides in either an α-(1→6) or α-(1→4) fashion. Additional sugar additions occurred that are hypothesized to be combinations of various linkage modes (α-(1→6) or α-(1→4)) of glucose. For example, the most abundant peak from two glucose additions (**3**) was determined to be α-(1→6); α-(1→6). Two other peaks from two glucose additions to linamarin have later elution times consistent with α-(1→4)-linked sugars, as the α-(1→6)-linked product (**1**) shows earlier elution than the α-(1→4)-linked product (**2**). The additional two peaks may therefore correspond to a compound with both α-(1→6) and α-(1→4)-linked sugars (**4**), and a compound with two α-(1→4) additions (**5**) as the last eluting peak. This is supported by the fact that the enzyme BtSUC5, which produces almost exclusively α-(1→4) glucosylated linamarin (**2**) in incubations with sucrose and linamarin, also produced **5** (Fig. 7b). Interestingly, the glucosylation of cyanogenic glycosides by *B. tabaci* resulted in the formation of many more peaks than the glucosylation of glucosinolates reported in our previous study^28^. However, regardless of whether linamarin had one or two α-glucose additions, these metabolites were resistant to activation by linamarase and thus represent detoxification products.

Phosphorylated linamarin glycosides were also detected in *B. tabaci* honeydew. Phosphorylation is hypothesized to be somewhat independent from the glucosylation of linamarin due to the capability of BtSUC2 and BtSUC5 to glucosylate both phosphorylated and non-phosphorylated linamarin (Supplemental Figure S7). Yet the biological role of phosphorylation is likely detoxification, similarly to glucosylation, as phosphorylated linamarin derivatives were not activated by linamarase. The formation of phosphorylated phenolic glycosides in *Lymantria dispar* (the gypsy moth) was also proposed to represent a detoxification reaction^39^. Although phosphorylation is an atypical mammalian phase II detoxification process, it may be more widespread in insects based on recent reports^40–42^. Phosphorylation of sugars occurs elsewhere in metabolism as a marker for breakdown, as in the case of glycolysis^43,44^, or in the synthesis of sugar polymers^44,45^. For example, C-3 phosphorylation as described here occurs in the process of glycogen synthesis^46,47^. The phosphorylation of plant glycosides in *L. dispar* and its close relative *Orgyia antigua* also occurred at C-3^39^.

The glucosylation of plant defense compounds is a logical detoxification reaction for phloem-feeding herbivores since phloem sap is rich in sugars. Moreover, phloem-feeding insects also possess transglucosidase enzymes that produce higher order sugar oligomers from sucrose in order to reduce the high osmotic pressures that may occur in the gut when feeding on phloem sap^48^. These enzymes can also glucosylate other substrates^28^, and were shown here in experiments with ^13^C-labeled sucrose isotopologues (Fig. 5) to be the enzymes involved in forming glucosylated cyanogenic glycoside derivatives. Two *B. tabaci* transglucosidases, BtSUC2 and BtSUC5, previously demonstrated to form glucosylated glucosinolates were shown to transglucosylate the cyanogenic glycoside linamarin in vitro. BtSUC2 produced the α-(1→6) (**1**) and α-(1→4) (**2**) linked monoglycosylated derivatives of linamarin identified in honeydew. In previous in vitro assays with these enzymes, derivatives with α-(1→6) linkages were not previously observed with glucosinolates as substrates^28^. BtSUC5 also displayed other products including a compound with a second glucose addition (**5**) that was not fully characterized. BtSUC5 is likely to also carry out the transglucosidation of linamarin in vivo since the *K*_M_ values for BtSUC5 were within physiologically expected levels for both linamarin and sucrose.

Further incubations of BtSUC2 and BtSUC5 with various plant defense glycosides resulted in the transglucosidation of virtually all substrates tested despite the great variety of aglycones present in the compounds offered, which included cyanogenic glycosides, glucosinolates, iridoid glycosides, benzoxazinoids, phenolic glycosides and flavonoid glycosides (Supplemental Figure S8). This substrate promiscuity suggests a very flexible catalytic pocket that accommodated all substrates, with the flavonoid glycoside rutin, the largest substrate tested, presumably fitting less well. A similar size limit on catalysis in transglucosideases was observed in studies with a dextran-sucrase where the velocity of reaction was reduced when switching the acceptor from maltose to the larger maltotriose^49^. The catalytic versatility of these enzymes in vitro was not observed in vivo when some of these substrates were fed directly to whiteflies^28^. Thus, more work needs to be carried out to determine the ability of these transglucosidases to react with plant defense compounds.

The glycoside hydrolase 13 family, which contains the transglucosidases, is greatly expanded in *B. tabaci* and other phloem-feeding insects^28,50^. The capacity of these enzymes to metabolize a wide range of plant defenses may be responsible for the broad host range of *B. tabaci*. In comparing the two *B. tabaci* species studied, the expression of the glycoside hydrolase gene *BtSUC5* was higher in BtSSA1-SG3 (which can feed on cassava but also on a wide range of alternative plant hosts)^32,36^ than in BtMEAM1 (a broad generalist not naturally found feeding on cassava) regardless of the host plant tested. Since at the protein level, BtSUC5 had a much higher transglucosidase activity than BtSUC2 with linamarin in vitro, the higher expression of the encoded gene *BtSUC5* in BtSSA1-SG3 vs. BtMEAM1 (Fig. 6c) may be important for the ability of this whitefly species to utilize cassava. The tissue-specific expression of *BtSUC2* and *BtSUC5* may also play an important role in this detoxification route. The expression of *BtSUC5* has been previously shown to be higher in the whitefly gut relative to its whole body^28^, however it is not currently known whether such enzymes are also produced in other tissues or might be secreted in the insect saliva to prevent hydrolysis before ingestion.

In conclusion, our investigations into *B. tabaci* whitefly metabolism of cyanogenic glycosides illustrate the diverse chemical transformations carried out by this phloem-feeding insect. Although cyanogenic glycosides and other two-component defenses were previously believed not to be activated by phloem-feeders, our results suggest that *B. tabaci* are indeed susceptible to two-component defenses and this may have selected for their potential to pre-emptively detoxify them by glucosylation and phosphorylation. Knowledge of how *B. tabaci* detoxifies cyanogenic glycosides could be employed to reduce its infestation of cassava, a cyanogenic glycoside-containing crop that is a staple food for millions of Africans. For example, RNA interference targeting the transglucosidases involved in detoxification could be applied via the cassava plant or by direct spraying^51^ to reduce whitefly feeding by increasing their susceptibility to cyanogenic glycosides. As this approach targets the whitefly specifically, it should not harm beneficial insects that come into contact with cassava. A parallel strategy could be to design specific inhibitors of the transglucosidase for application to the crop. Further information on the metabolic adaptations of whiteflies to cassava could also be exploited in crop protection and contribute additionally to basic knowledge on how phloem-feeding insects survive on plants with two-component defenses.

## Materials and methods

### Plants

Eggplant (*Solanum melongena*, cv. Black Beauty), and cassava (*Manihot esculenta*, cv. MCol22) plants were grown under standard greenhouse conditions at 26 ± 2 °C with supplemental lighting and a photoperiod of 14:10 h (light:dark).

### Insects

*Bemisia tabaci* (Hemiptera: Aleyrodidae) species MEAM1 (Middle East-Asia Minor 1) was collected in southern Israel in 2003 and from Sudan in the late 1990s, and reared continuously on cotton as separate populations. An additional MEAM1 population was collected in Peru in 2012 and maintained on eggplant. *B. tabaci* species SSA1-SG3 (sub-Saharan Africa 1—species group 3) was collected on Bagamoyo Road, Tanzania in 2013 and reared on cassava plants.

### *Bemisia tabaci* feeding on cassava and eggplant for beta-cyanoalanine analysis

Groups of adult *B. tabaci* (200 individuals, BtSSA1-SG3), were reared on eggplant or cassava, then collected 1-3 days after emergence and kept frozen prior to extraction and LC-MS analysis of beta-cyanoalanine.

### *Bemisia tabaci* feeding on cassava and eggplant for analysis of *BtSUC2* and *5* expression

Groups of adult *B. tabaci* (50 individuals of BtSSA1-SG3 and BtMEAM1), were reared on sucrose-based artificial diets for 72 h and transferred to cassava or eggplant for 24 h. Further RNA isolation and analysis was conducted as in^32^, and gene expression analyses were performed as described below.

### Honeydew collection

Honeydew was collected from three different sources:

### *Bemisia tabaci* feeding on cassava for honeydew metabolite analysis

Groups of adult *B. tabaci* (50 individuals, BtSSA1-SG3), were reared on eggplant and transferred 1–3 days after emergence to fresh cassava. Insects were enclosed within glass clip cages while feeding After 96 h, the honeydew deposited on the glass tubes was washed off with water:methanol (20:80, v:v), dried under nitrogen gas, and resuspended in water prior to LC–MS analysis of phosphorylated and glucosylated linamarin derivatives.

### *Bemisia tabaci* feeding on artificial diets with linamarin

Feeding of artificial was replicated as described in^28^ with changes to diet composition and collection methods as described below. Groups of 150 *B. tabaci* (MEAM1) adults were collected from eggplant and switched to artificial diet feeders consisting of a glass tube (3 cm height × 2 cm diameter) with a liquid diet covered with a double layer of Parafilm. Insects were allowed to feed through the Parafilm on a 10% sucrose solution containing no additives (control) or the cyanogenic glycoside linamarin (Sigma-Aldrich) at a concentration of 5 mM. After 96 h, the honeydew deposited on the glass tubes was washed off with water:methanol (20:80, v:v), dried under nitrogen gas, and resuspended in water prior to LC–MS analysis of phosphorylated and glucosylated linamarin derivatives.

### *Bemisia tabaci* feeding on artificial diets containing isotopically-labeled sugars and linamarin

Feeding of isotopically labelled sugars was performed as in^28^ with diet composition modified accordingly. Four different sucrose isotopologues were added to artificial diets: [^12^C_12_]sucrose, [^13^C_12_]sucrose, [glucose-^13^C_6_]sucrose, and [fructose-^13^C_6_]sucrose. The monosaccharides [^13^C_6_]fructose and [^13^C_6_]glucose were also fed. The artificial feeding devices consisted of a glass tube (5 cm high × 2.5 cm diameter) with the liquid diet (50 µL) held within a double layer of Parafilm. About 50 *B. tabaci* MEAM1 adults were placed in each tube. Feeding assays were performed for 72 h on diets that contained 5 mM linamarin and 0.29 M of the labeled sugars. The honeydew deposited on the glass tubes was washed with water: methanol (20:80, v:v) and stored at − 20 °C until processing and further MS analysis of ^13^C isotope incorporation into linamarin-derived glucosylated metabolites. A full summary of artificial diet constituents is outlined in Supplementary Table S2.

### Purification and LC–MS analysis of glucosylated and phosphorylated cyanogenic glycosides

Purification of glucosylated or phosphorylated cyanogenic glycosides was performed similarly to^28^, via fractionation on a Nucleodur Sphinx RP column (250 × 4.6 mm, 5 µm, Macherey–Nagel, Düren, Germany) using an HP 1200 HPLC (Agilent Technologies, Santa Clara, CA, USA) coupled to a fraction collector (Advantec, Dublin, CA, USA), with methods modified accordingly. Chromatographic separation was attained using a gradient of 0.05% aqueous formic acid (Solvent A) and acetonitrile (Solvent B) at a flow rate of 1 mL min^−1^ at 20 °C as follows: 5–29% B (12 min), 29–100% B (0.1 min), a 2.9 min hold at 100% B, 100–5% B (0.1 min), and a 3.9 min hold at 5% B. Linustatin was purified from linseed by crushing 10 g of seeds in liquid nitrogen and extracting with 80% methanol prior to centrifugation at 10,000×*g*. The methanol supernatant was then dried down and concentrated before resuspension in water for purification on a reverse phase column as described above.

Qualitative analysis of glucosylated cyanogenic glycosides in feces and honeydew extracts was performed on an HP 1100 series HPLC as in^28^ using instrument parameters modified accordingly, as described below. Separation was achieved on a Nucleodur Sphinx RP column (250 × 4.6 mm, 5 µm, Macherey–Nagel) with a gradient of 0.2% aqueous formic acid (solvent A) and acetonitrile (solvent B) with a flow rate of 1 mL min^−1^ at 25 °C as follows: 5–55% B (25 min), 55–100% B (0.1 min), 100% B 0.9 min hold, 100–5% B (0.1 min), 5% B 3.9 min hold. The HPLC was coupled to an Esquire 6000 ESI-Ion Trap mass spectrometer (Bruker Daltonics, Bremen, Germany) operated in both positive and negative modes in the range of *m*/*z* 60–1500 with skimmer voltage − 40 V; capillary exit voltage − 146.7 V; capillary voltage 4000 V; nebulizer pressure 35 psi; drying gas 11 L min^−1^; and gas temperature 330 °C. DataAnalysis software V4 (Bruker Daltonics) was used for chromatogram analysis. Qualitative analysis of isotopically-labeled glucosylated cyanogenic glycosides in feces and honeydew extracts was performed by LC–MS as described above for feces and honeydew except that capillary exit volatage was − 113.5 V.

High resolution mass spectrometry of phosphorylated compounds was achieved on an Thermo Scientific UltiMate 3000 UHPLC coupled to a Bruker TIMS-TOF mass spectrometer. Separation was achieved on a Nucleodur Sphinx RP column (250 × 4.6 mm, 5 µm, Macherey–Nagel, Germany) with a gradient of 0.2% aqueous formic acid (solvent A) and acetonitrile (solvent B) with a flow rate of 1 mL min^−1^ (split 1:3 source: waste) at 25 °C as follows: 5–55% B (25 min), 55–100% B (0.1 min), 100% B 0.9 min hold, 100–5% B (0.1 min), 5% B 3.9 min hold. The MS was operated in negative mode scanning from *m/z* 50–1500 with the following parameters. Source End plate offset: 500 V, capillary: 3500 V, Neubilizer: 3.5 bar, Dry gas: 11.0 L min^−1^, Dry temperature 330 °C. Tune General Funnel 1RF: 150 Vpp, Funnel 2 RF: 200 Vpp, isCID energy: 0.0 eV, Multipole RF: 50 Vpp, Deflection Delta: − 70 V, Quadrupole energy: 4.0 eV, Low mass: 90 m*/z* Collision energy: 7.0 eV, Collision RF: 400 Vpp, Transfer time: 80.0 µs, Pre-pulse storage: 5.0 µs. Calibration took place externally immediately before the samples were run using Agilent ESI-L Low Concentration Tune Mix and an enhanced quadratic calibration curve.

Quantification of the glucosylated cyanogenic glycosides in transglucosidase and linamarase/linustatinase assays and in honeydew was accomplished via an HP 1260 series HPLC coupled to an AB Sciex API 5000 mass spectrometer (Applied Biosystems, Darmstadt, Germany). The column utilized was a Nucleodur Sphinx RP column (250 × 4.6 mm, 5 µm, Macherey–Nagel) using a chromatographic gradient of 0.05% aqueous formic acid (Solvent A) and acetonitrile (Solvent B) at a flow rate of 1 mL min^−1^ at 20 °C as follows: 5–29% B (12 min), 29–100% B (0.1 min), a 2.9 min hold at 100% B, 100–5% B (0.1 min), and a 3.9 min hold at 5% B.The MS was operated in the negative mode with collision gas value 7, curtain gas pressure 35 psi, spray gas pressures 60 psi, ion spray voltage − 4500 V, and turbogas temperature 600 °C. Compounds were detected using multiple reaction monitoring (MRM) detection with the parameters outlined in Supplementary Table S3. Quantification was achieved using external calibration curves constructed from solutions of purified glucosylated cyanogenic glycosides of known concentrations (determined in solution via NMR as described below). Analyst 1.5 software (Applied Biosystems) was used for data acquisition and processing. All averages and standard errors were calculated from three independent biological replicates. No other statistical tests were performed.

### Extraction and LC–MS analysis of beta-cyanolalanine

Groups of 200 *B.* *tabaci* adults (BtSSA1-SG3) were collected from eggplant and cassava plants. The bodies were crushed using liquid nitrogen and extracted in 1 mL of water:methanol (20:80, v:v). The supernatant was evaporated using nitrogen and resuspended in 50 µL water before LC–MS analysis (see below).

For analysis of beta-cyanoalanine in plants, 3 young leaves of cassava and eggplant each were crushed using liquid nitrogen and a mortar and pestle and extracted in 0.5 mL water:methanol (20:80, v:v) per mg of leaf tissue. Aliquots of the supernatants were then evaporated and resuspended in identical volumes of water before LC–MS analysis.

Quantification of beta-cyanoalanine levels was performed on an HP 1260 HPLC coupled to an AB Sciex API 5000 mass spectrometer. The column utilized was a Agilent XDB-C18 column (50 × 4.6 mm, 1.8 µm, Agilent Technologies, Boeblingen, Germany) using a chromatographic gradient of 0.05% aqueous formic acid (Solvent A) and acetonitrile (Solvent B) with a flow rate of 1.1 mL min^−1^ at 25 °C as follows: 0.5 min hold at 10% B, 10–45% B (3.5 min), 45–100% B (0.02 min), 0.98 min hold at 100% B, 100–10% B (0.02 min), 1.98 min hold at 10% B. The mass spectrometer was operated in the negative mode with collision gas value 8, curtain gas pressure 25 psi, spray gas pressures 60 psi, ion spray voltage − 4500 V, and turbogas temperature 700 °C. Compounds were detected using scheduled multiple reaction monitoring (MRM) detection with the parameters outlined in Supplementary Table S3. Analyst 1.5 software was used for data acquisition and processing.

### NMR spectroscopy

NMR analyses were performed as in^28^, with spectra (^1^H, ^1^H-^1^H COSY, ^1^H-^13^C HSQC, ^1^H-^13^C HMBC and ^1^H-^1^H SELTOCSY) acquired on a 700 MHz Avance III HD spectrometer equipped with a 1.7 mm cryoprobe (Bruker Biospin, Rheinstetten, Germany). Further information is available in the supplementary information file. Data acquisition and processing was accomplished using TopSpin ver. 3.2 (Bruker Biospin, Rheinstetten, Germany). Samples were measured in MeOH-*d*_3_ or D_2_O as indicated at 293 K. For quantification via ^1^H NMR (10 s delay between scans), purified compounds were dried under N_2_ flow and resuspended in D_2_O, and a sucrose solution (3.13 mM in D_2_O) was used as an external quantification standard.

### Cloning and heterologous expression of *B. tabaci* transglucosidases in *Drosophila* S2 cells

Vectors, cells and cell media were obtained from ThermoFisher Scientific (Waltham, MA, USA). Full-length ORFs from previously characterized *B. tabaci* Glucohydrolase Family 13 genes (*BtSUC2* and *BtSUC5*) were amplified as previously described^28^ using the primer sets outlined in Supplemental Table S4.

### Determination of *BtSUC2* and *BtSUC5* gene expression

The number of raw reads mapping to *BtSUC2* and *BtSUC5* were determined from SRP127757^32,36^ using the STAR software^52^, based on the genome and gtf files available at NCBI. Sequence alignment between SSA1 and MEAM1 sequences was performed using the BtSSA1 genome (unpublished, provided by EBI).

### Transglucosidase enzyme assays

Enzyme assays were performed as in^28^ with modifications listed below. *Drosophila* S2 cells expressing BtSUC2 and BtSUC5 enzymes as well as non-transfected control cells were centrifuged at 100×*g* for 5 min. The resulting supernatant was utilized as the secreted protein fraction for enzyme assays. For determination of linamarin transglucosidation activity, 5 µL of supernatant from each culture containing an equivalent total protein content (verified by Bradford assays) was mixed with a 5 µL 50 mM phosphate buffer at pH 7.0 containing 2 M sucrose and 5 mM linamarin, and reacted for 6 h at 25 °C with no stirring. These assay conditions were also repeated for other potential substrates: I3M-GSL (Phytoplan Diehm & Neuberger GmbH), pOHBz-GSL(Phytoplan), DIMBOA-Glc, amygdalin (Roth), dhurrin (Roth), salicin (Sigma), arbutin (Roth), aucubin (Roth), and rutin (Sigma). Assay conditions were chosen after screening a range of substrate concentrations and pH values that reflect previous work on glucohydrolases and phloem-feeding insects. The reaction was stopped using 15 μL of methanol and immediately stored at − 20 °C. For LC–MS analysis, enzyme assays were centrifuged at 5200×*g* for 5 min and the supernatant obtained was diluted 1:10 in water for the analysis of glucosylated linamarin, and diluted 1:1000 in water.

For *K*_M_ estimation, enzyme preparations of control cells were first checked and found to catalyze less than 1% of the supplied linamarin to transglucosylated products also to catalyze only low levels of sucrose hydrolysis. Enzyme assays were performed in the same manner as described above, except incubation times were reduced to 30 min. Peak areas were integrated and compared based on duplicate analysis. The sucrose *K*_M_ determination was performed at a constant linamarin concentration of 2.5 mM with sucrose concentrations ranging from 0.05 to 1 M. The linamarin *K*_M_ determination utilized sucrose at a final concentration of 1 M and a range of linamarin concentrations from 25 µM to 20 mM. All assays were carried out under linear reaction conditions with respect to time and protein concentration. Substrate concentration was never reduced below 95% of the initial level during the 30 min assay period.

For tests on the reactivity of glucosylated cyanogenic glycosides with plant linamarase, linamarin (Sigma) (10 µL of a 5 mM solution), linustatin purified from linseed extracts (see above section on cyanogenic glycoside purification) and honeydew from cassava-reared adult SSA1-SG3 whiteflies containing glucosylated linamarin glycosides were mixed with a crude leaf enzyme extract from cassava with native linamarase activity or crude linseed extracts with native linustatinase activity in 20 mM phosphate buffer solution (10 μL), pH 7.0. The cassava linamarase extract was obtained from 10 g of leaves and the linseed linustatinase extract was obtained from 10 g of seeds flash frozen in liquid nitrogen, crushed using a mortar and pestle, extracted with phosphate buffer (20 mM pH 7.0) and filtered using vacuum filtration. Control reactions were supplemented with 5 µL of water instead of the cassava or linseed extract solutions. Reactions were incubated at room temperature with no stirring, stopped after 1 h with 20 µL acetic acid and stored frozen until LC–MS analysis. Phosphorylated derivatives were tested for stability to cassava crude enzyme extract (but not to linseed extract) under the same reaction conditions.

## Supplementary Information

Supplementary Information.
