# Supplementary material for: Activation and detoxification of cassava cyanogenic glucosides by the whitefly Bemisia tabaci
Source: Sci Rep. 2021 Jun 24;11:13244. doi: 10.1038/s41598-021-92553-w (PMC8225905; doi:10.1038/s41598-021-92553-w)
Supplement: Supplementary file 1 — Supplementary Information. [file 41598_2021_92553_MOESM1_ESM.docx]

**Supplementary Information**

**Activation and detoxification of cassava cyanogenic glucosides by the whitefly *Bemisia tabaci***

Michael L.A.E. Easson^1^, Osnat Malka^2^, Christian Paetz^1^, Anna Hojná^1^, Michael Reichelt^1^, Beate Stein^3^, Sharon van Brunschot^4,5^, Ester Feldmesser^6^, Lahcen Campbell^7^, John Colvin^4^, Stephan Winter^3^, Shai Morin^2^, Jonathan Gershenzon^1^, Daniel G. Vassão^1^

*^1^Max Planck Institute for Chemical Ecology, Jena 07745, Germany*

*^2^The Hebrew University of Jerusalem, Rehovot 7610001, Israel*

*^3^Leibniz Institute DSMZ-German Collection of Microorganisms and Cell Cultures, Braunschweig 38104, Germany*

*^4^Natural Resources Institute, University of Greenwich, Chatham Maritime, Kent ME4 4TB, UK*

*^5^University of Queensland, Brisbane, Queensland 4072, Australia*

*^6^Weizmann Institute of Science, Rehovot 7610001, Israel*

*^7^EMBL-European Bioinformatics Institute*, *Cambridge CB10 1SD, UK*

Corresponding authors:

*Daniel G. Vassão: vassao@ice.mpg.de*

*Osnat Malka: osnat226@gmail.com*

**Supplemental Figures**


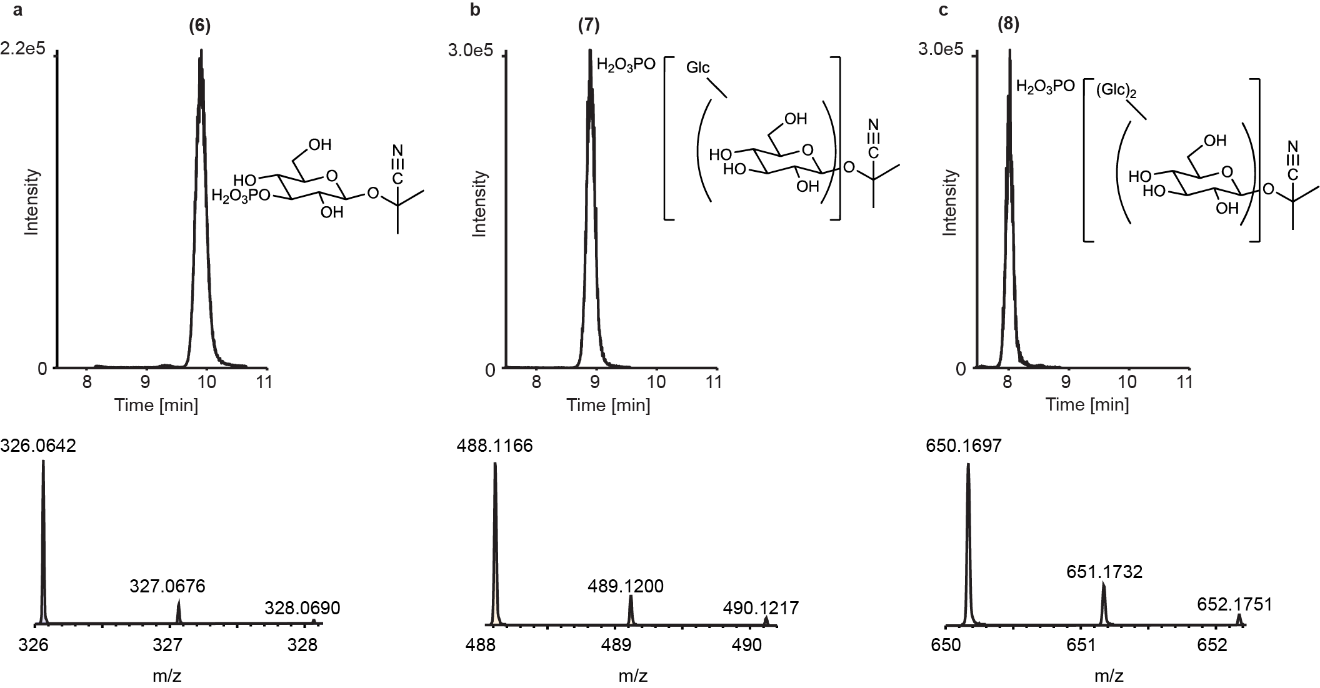


**Figure S1: HRMS supports the addition of a phosphate.** HRMS data of a honeydew sample showing mass spectra and retention times for compounds **(6, 7 and 8)** (panels a, b and c respectively).


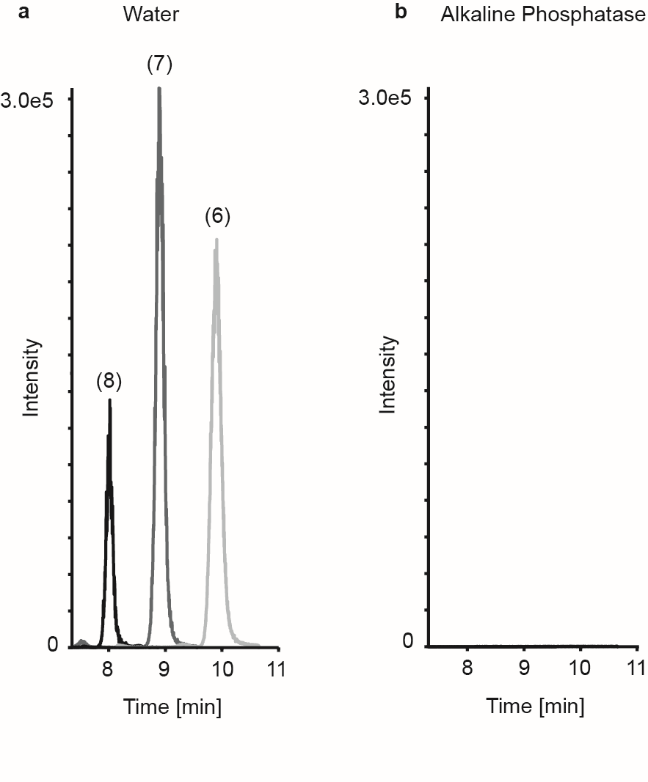


**Figure S2: Alkaline phosphatase incubation results in the disappearance of mass signals for phopshorylated compounds**. Phosphorylated compounds **(6, 7 and 8)** in the honeydew of the whitefly feeding on cassava were incubated with water (a) or alkaline phosphatase (b). The disappearance of the mass signals supports the compounds being phosphorylated products.


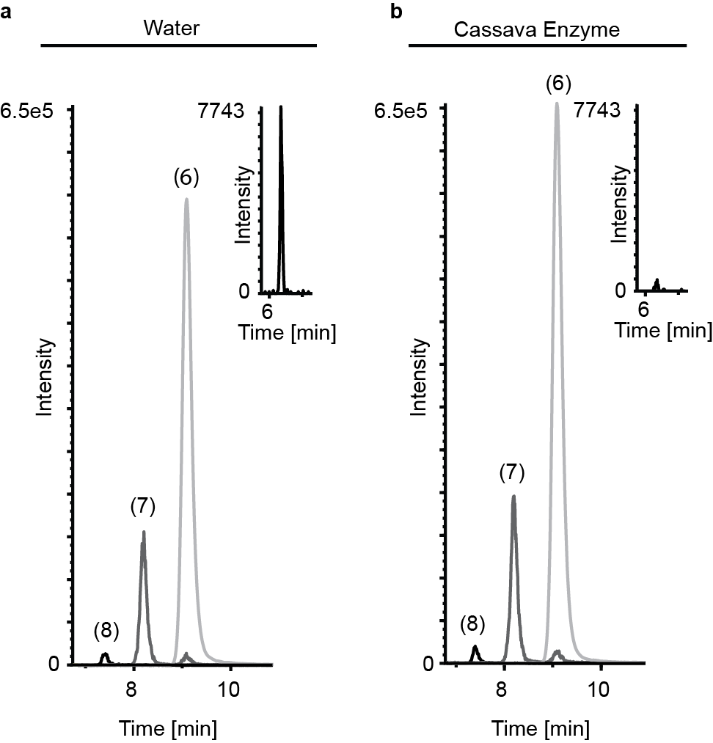


**Figure S3: Phosphorylated linamarin metabolites in *B. tabaci* honeydew are resistant to linamarase activation.** Phosphorylated metabolites **(6, 7 and 8)** in honeydew were incubated with water (**a**) and crude cassava leaf enzymes containing linamarase activity (**b**). Compounds (**6**), (**7**) and (**8**) were resistant to activation by cassava linamarase, while the parent linamarin is readily hydrolyzed. Samples were incubated for 1 h with cassava crude enzyme extract or water in phosphate buffer at pH 7.0.


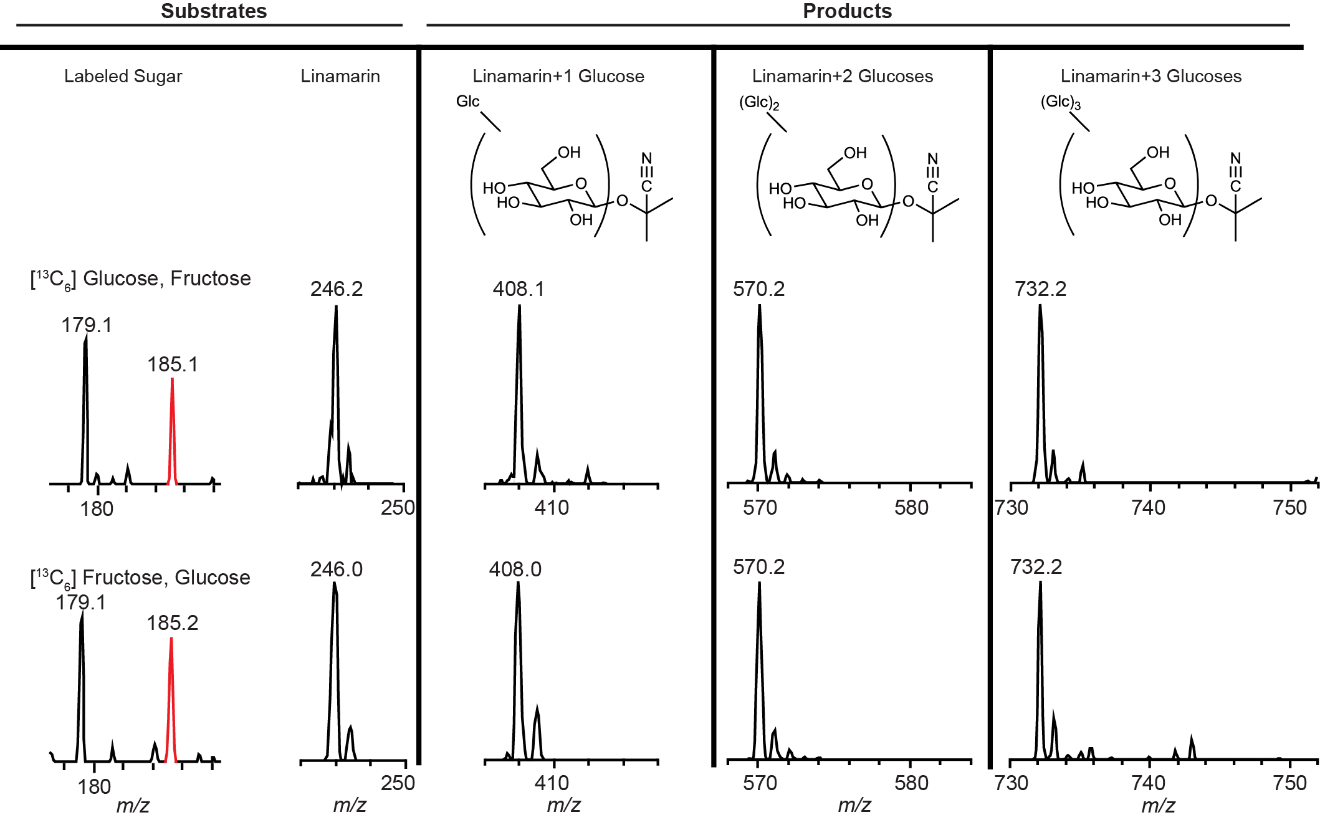


**Figure S4:** **Linamarin glucosylation in the whitefly *B. tabaci* is catalyzed by a transglucosidase activity.** Depiction of the results from two of the five diets not shown in Figure 4, those diets with the ^13^C-labeled monosaccharides glucose and fructose. None gave labeled glycosylated linamarin products, unlike feeding with sucrose labeled in the glucose portion. The results are consistent with a transglucosidase activity that initially hydrolyzes sucrose and links the resulting glucose moiety to linamarin.


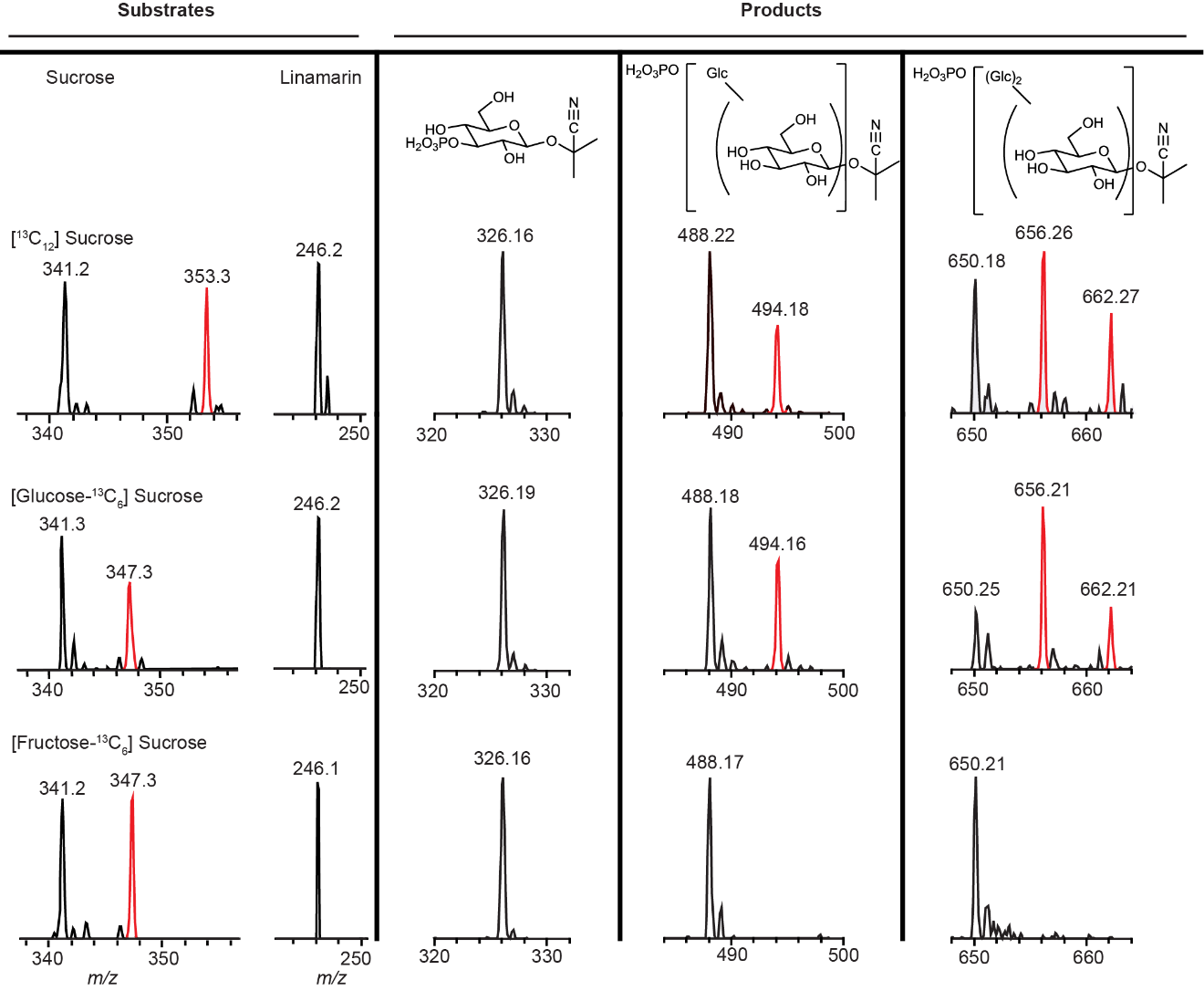


**Figure S5: Glucosylation of phosphorylated linamarin derivatives is also catalyzed by a transglucosidase.** Shown are regions of mass spectra from LC-MS analyses of substrates and products of feeding experiments on three different diets. Feeding fully ^13^C-labeled sucrose and sucrose ^13^C-labeled in the glucose moiety gave labeling in the newly added glucose(s) of linamarin phosphate derivatives (**7 and 8**).


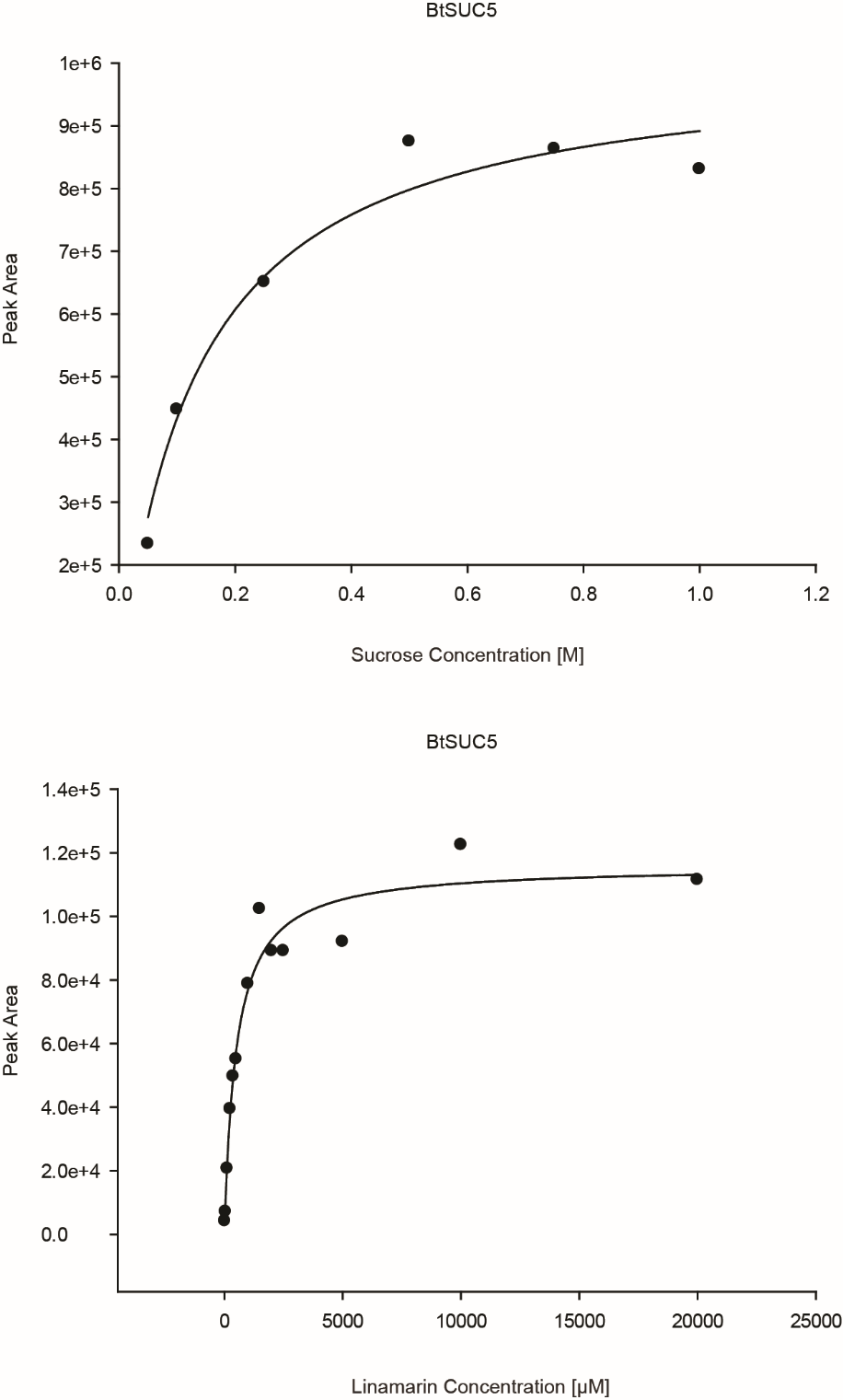


**Figure S6:** **Kinetic characterization of BtSUC5 with linamarin and sucrose**. Depicted are the relations between substrate concentration and reaction rate (product peak area) for both sucrose and linamarin. Assays were carried out as described in the methods section. When the sucrose concentration was varied from 0.05 to 1.0 M, linamarin concentration was held at 2.5 mM. When the linamarin concentration was varied from 25 µM to 20 mM, sucrose concentration was held at 1 M.


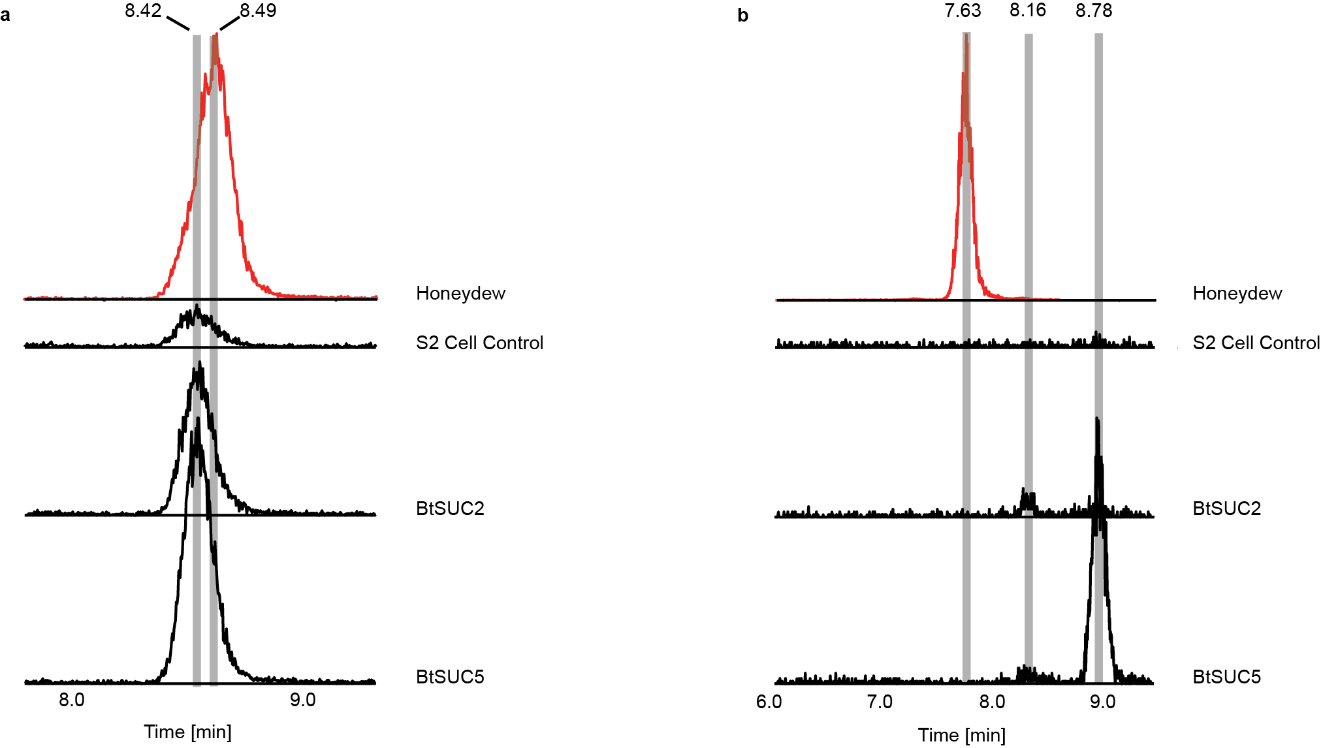


**Figure S7: Chromatographic analyses of products from BtSUC2 and 5 enzymes heterologously produced in *D. melanogaster* S2 cells.** Cell medium activity was assayed with phoshorylated linamarin derivatives and sucrose. (**a**) Depicted are extracted multiple reaction monitoring (MRM) LC-MS chromatograms for **(7)** upon incubating **(6)** with sucrose and enzymes BtSUC2 and BtSUC5. The enzymes BtSUC2 and 5 showed transglucosidation activity, producing a glycosylated derivative of **(6)** however the retention differed from that of the **(7)** found within the honeydew. (**b**) Repeating the assay with purified **(7)** as a starting substrate, enzyme BtSUC2 produced low levels of a glycoside of **(7)** but again differing from honeydew peaks and BtSUC5 produced a glycoside of even greater retention difference. S2 cell control assay was of cell medium extracts of untransformed cells.


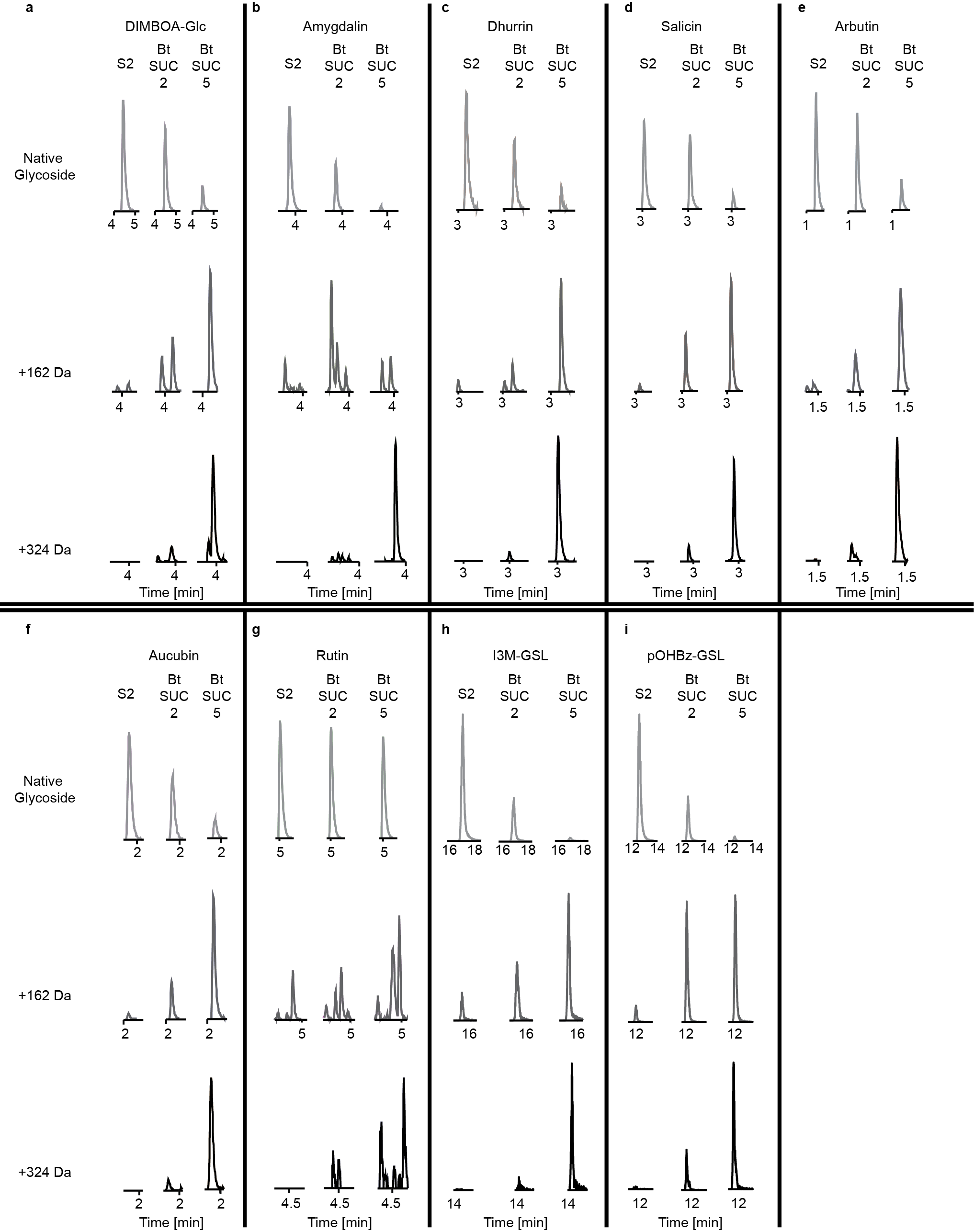


**Figure S8: Substrate tests for BtMEAM1 BtSUC2 and 5 enzymes heterologously produced in *D. melanogaster* S2 cells.** Cell medium activity was assayed with various secondary metabolites and sucrose. Depicted are masses for the native glycoside and the addition of one glucose (+162 Da) and 2 glucose units (+324 Da). BtSUC2 and BtSUC5 showed transglucosidation activity with all substrates above control levels, with rutin poorest glucose acceptor based on native glycoside peak depletion. Compounds used were (**a**) DIMBOA-Glucose (benzoxazinoid), (**b**) amygdalin (cyanogenic diglycoside), (**c**) dhurrin (cyanogenic mono-glycoside), (**d**) salicin (phenolic glycoside), (**e**) arbutin (phenolic glycoside), (**f**) aucubin (irridoid glycoside), (**g**) rutin (flavanoid diglycoside), (**h**) indoyl-3-methyl glucosinolate (glucosinolate), and (**i**) para-hydroxybenzyl glucosinolate (glucosinolate). S2 cell control assay was of cell medium extracts of untransformed cells. All peaks are normalized within each set of assays to the largest peak of the respective metabolite.

**Supplementary Tables**

**Table S1: HRMS data for phosphorylated linamarin derivatives showing measured and expected masses of the metabolites.**


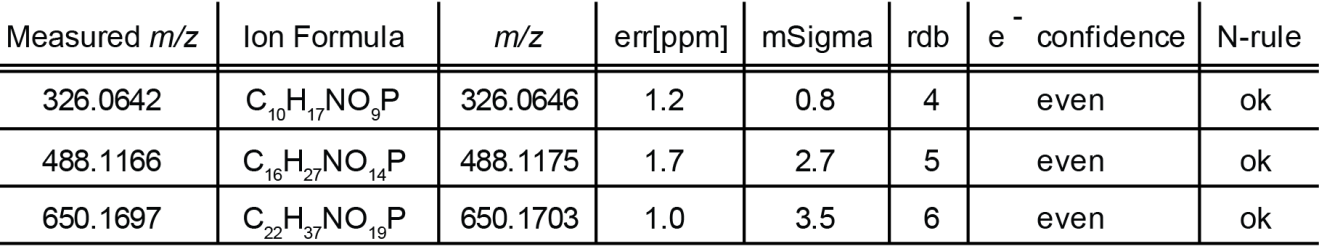


**Table S2: Constituents of artificial diets fed to *Bemisia tabaci* MEAM1.**


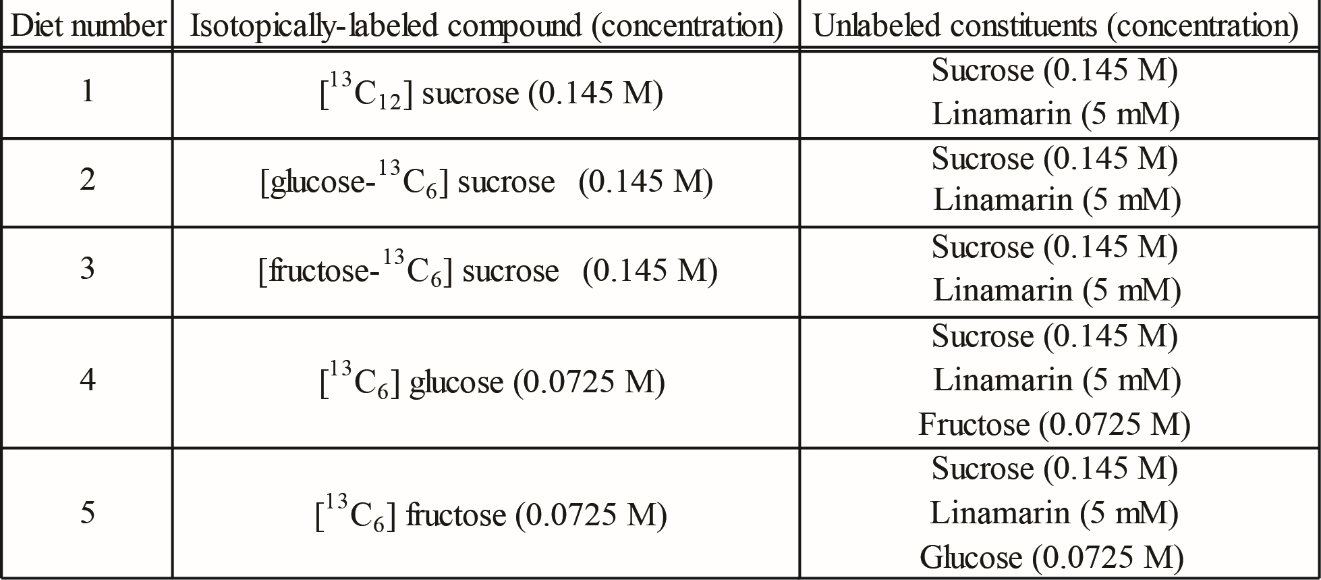


**Table S3: List of multiple reaction monitoring (MRM) parameters for individual compounds analyzed by LC-MS.**


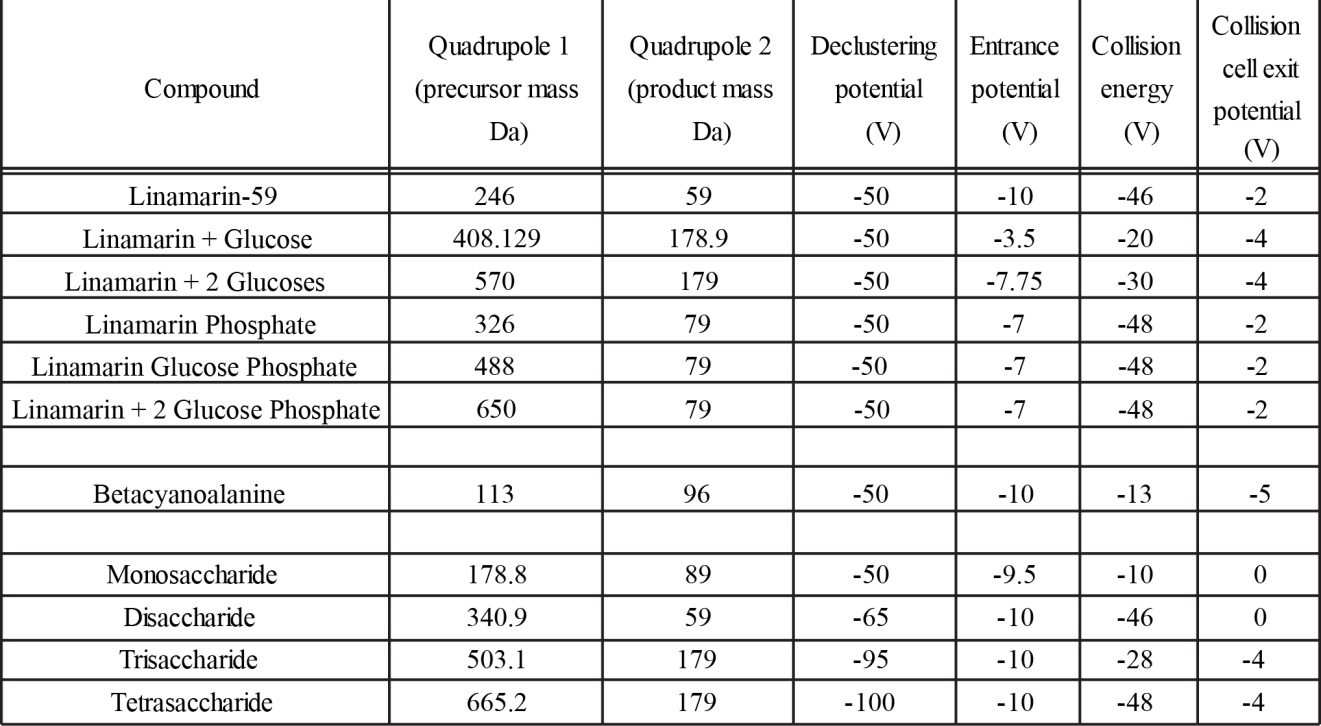


**Table S4: List of primers used for cloning of GH13 genes from *B. tabaci***


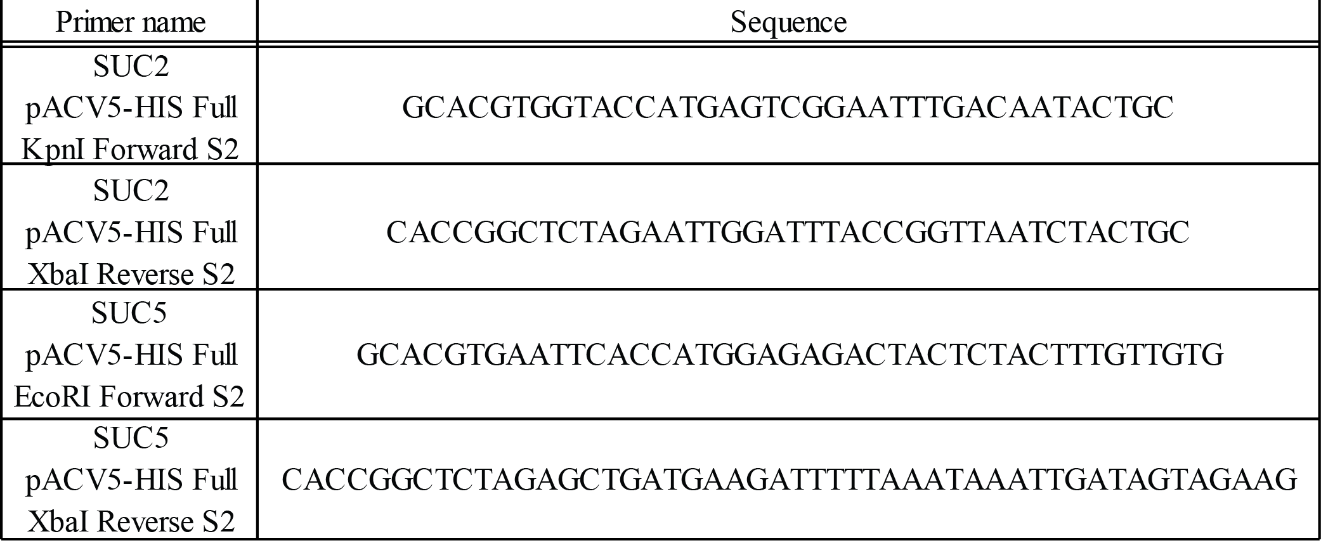


**Supplementary Note 1**

**Supporting information NMR**

NMR spectra were recorded on a Bruker Avance III HD 700 MHz spectrometer, equipped with a cryoplatform and a 1.7 mm cryoprobe (Bruker Biospin GmbH, Rheinstetten, Germany). Spectrometer control and data processing was accomplished using Bruker TopSpin ver. 3.2. ^13^C chemical shifts were determined indirectly by means of ^1^H-^13^C HSQC (heteronuclear single quantum coherence) and ^1^H-^13^C HMBC (heteronuclear multiple bond correlation) experiments. The glucosidic substitutions were elucidated with the help of 1D ^1^H-^1^H SELTOCSY (selective total correlation spectroscopy) experiments. The resulting spectra served as projection spectra for the 2D homo- and heteronuclear experiments. The positions of the chemical shifts in the molecules were furthermore determined based on ^3^JHH coupling constants and ^13^C chemical shifts. Important spectral details have been assembled for deeper understanding of the structure elucidation as follows.

**Figure SN1-1:** Structure of **2** with chemical shifts in D_2_O

**Table SN1-1:** Table of chemical shifts of **1, 2** and **3** in D_2_O

**Figure SN1-2:** Structure of **2** with chemical shifts in D_2_O

**Figure SN1-3:** Structure of **1** with chemical shifts in D_2_O

**Figure SN1-4:** Structure of **3** with chemical shifts in D_2_O

**Figure SN1-5:** Table of chemical shifts of **6** in D_2_O with numbered structure

**Figure SN1-6:** Structure of **6** with chemical shifts in D_2_O

**Figure SN1-7:** 1dNOESY (black trace) and a SELTOCSY spectrum of **6**

**Figure SN1-8:** DQFCOSY of **6**

**Figure SN1-9:** ^1^H-^13^C HSQC spectrum of **6**, glucosidic range

**Figure SN1-10:** ^1^H-^13^C HSQC spectrum of **6**, CH_3_ range

**Figure SN1-11:** ^1^H-^13^C HSQC spectrum of linamarin in MeOH-*d_3_*


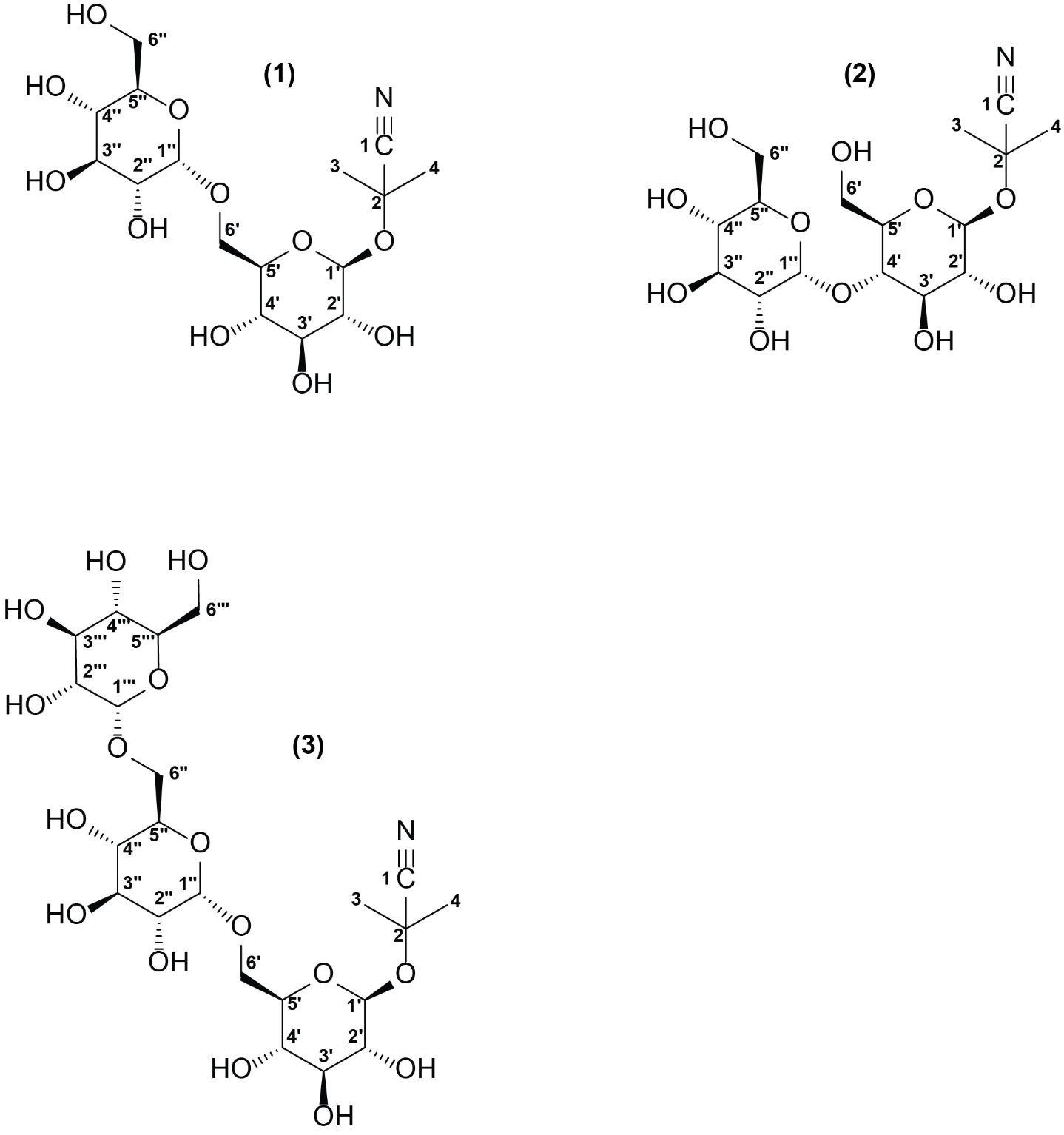


**Figure SN1-1:** Structure of glucosylated linamarin derivatives (compounds **1**, **2** and **3**) with numbering.

**Table SN1-1: ^1^H and ^13^C NMR shifts and coupling constants for glucosylated linamarin derivatives.**

**
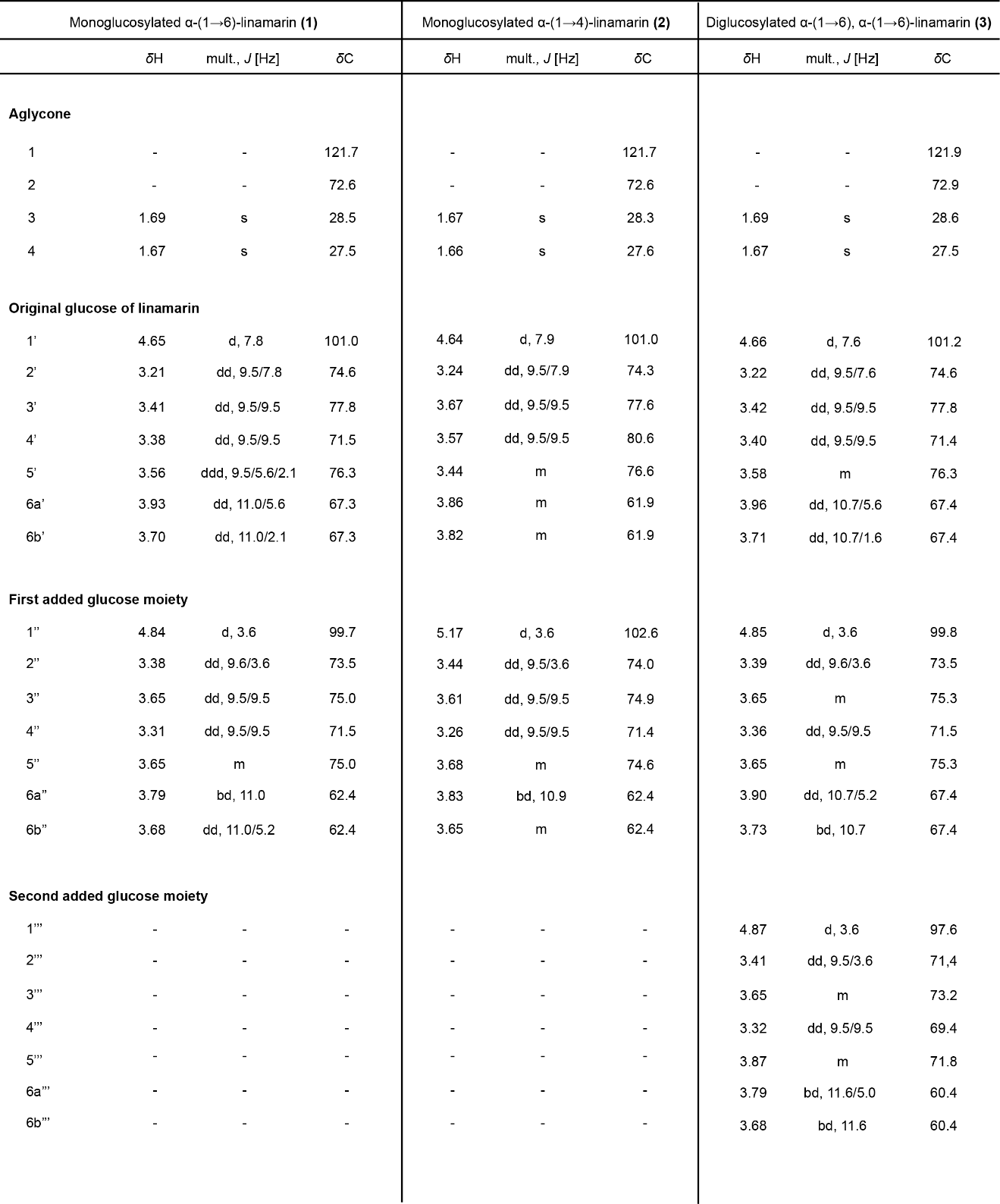
**


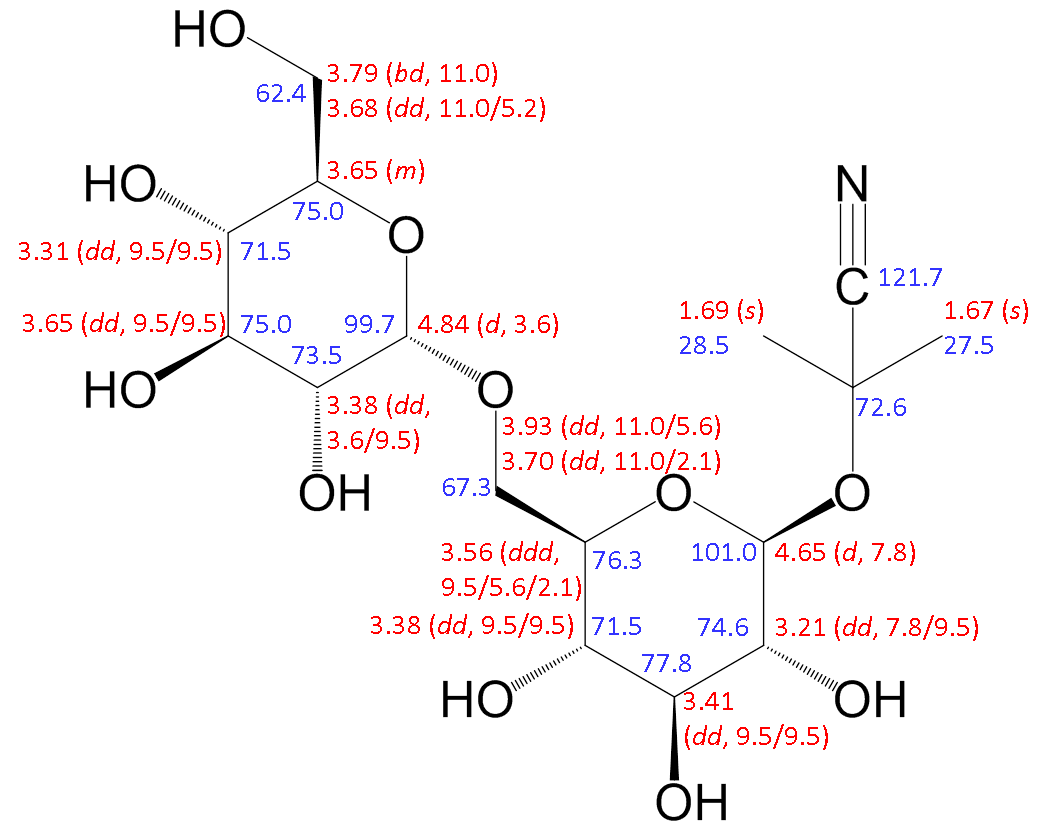


**Figure SN1-2:** Structure and chemical shifts of monoglucosylated α-(1→6)-linamarin (**1**). ^1^H chemical shifts in red, ^13^C chemical shift in blue.


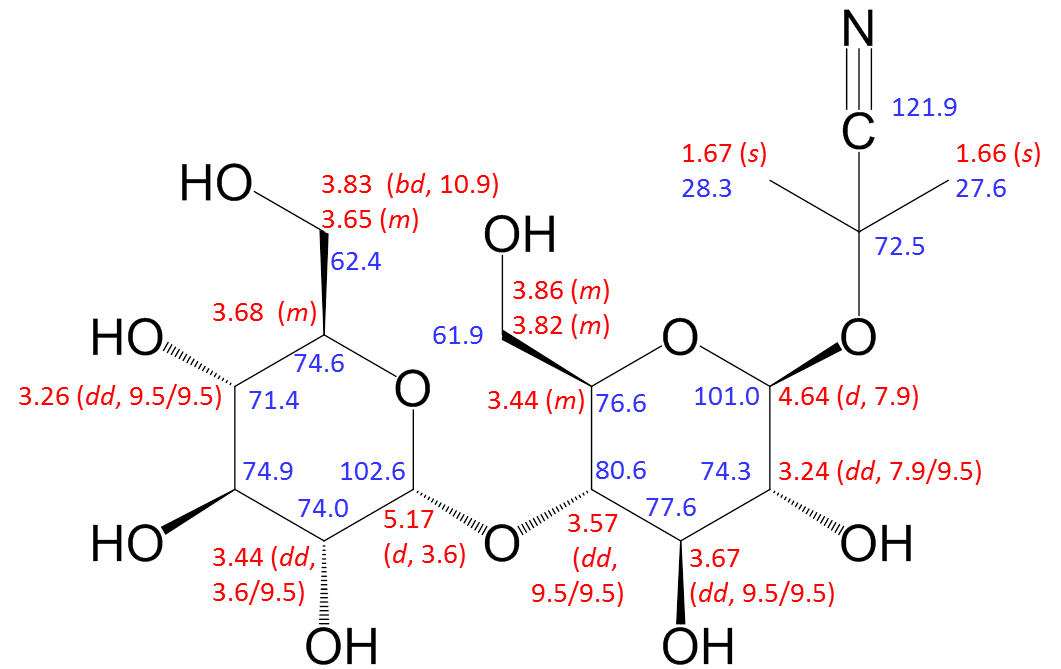


**Figure SN1-3:** Structure and chemical shifts of monoglucosylated α-(1→4)-linamarin (**2**). ^1^H chemical shifts in red, ^13^C chemical shift in blue.


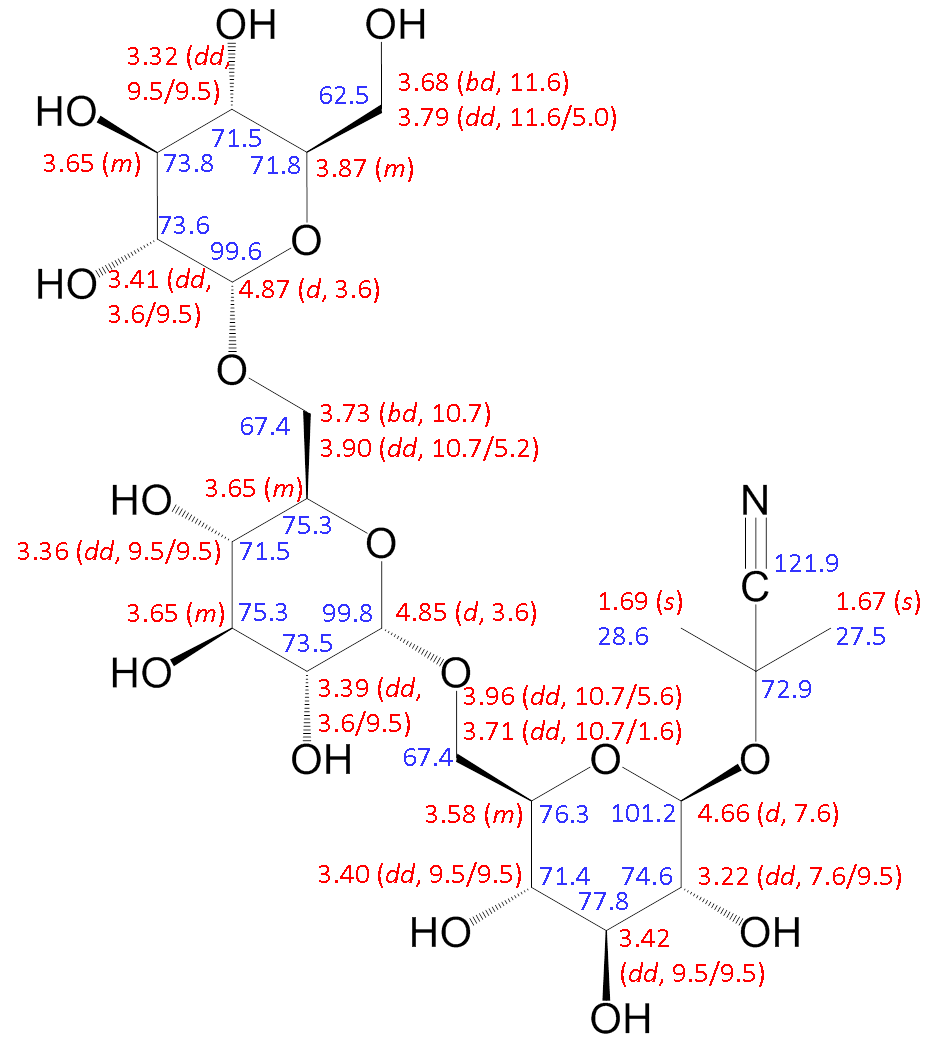


**Figure SN1-4:** Structure and chemical shifts of diglucosylated α-(1→6), α-(1→6)-linamarin (**3**). ^1^H chemical shifts in red, ^13^C chemical shift in blue.


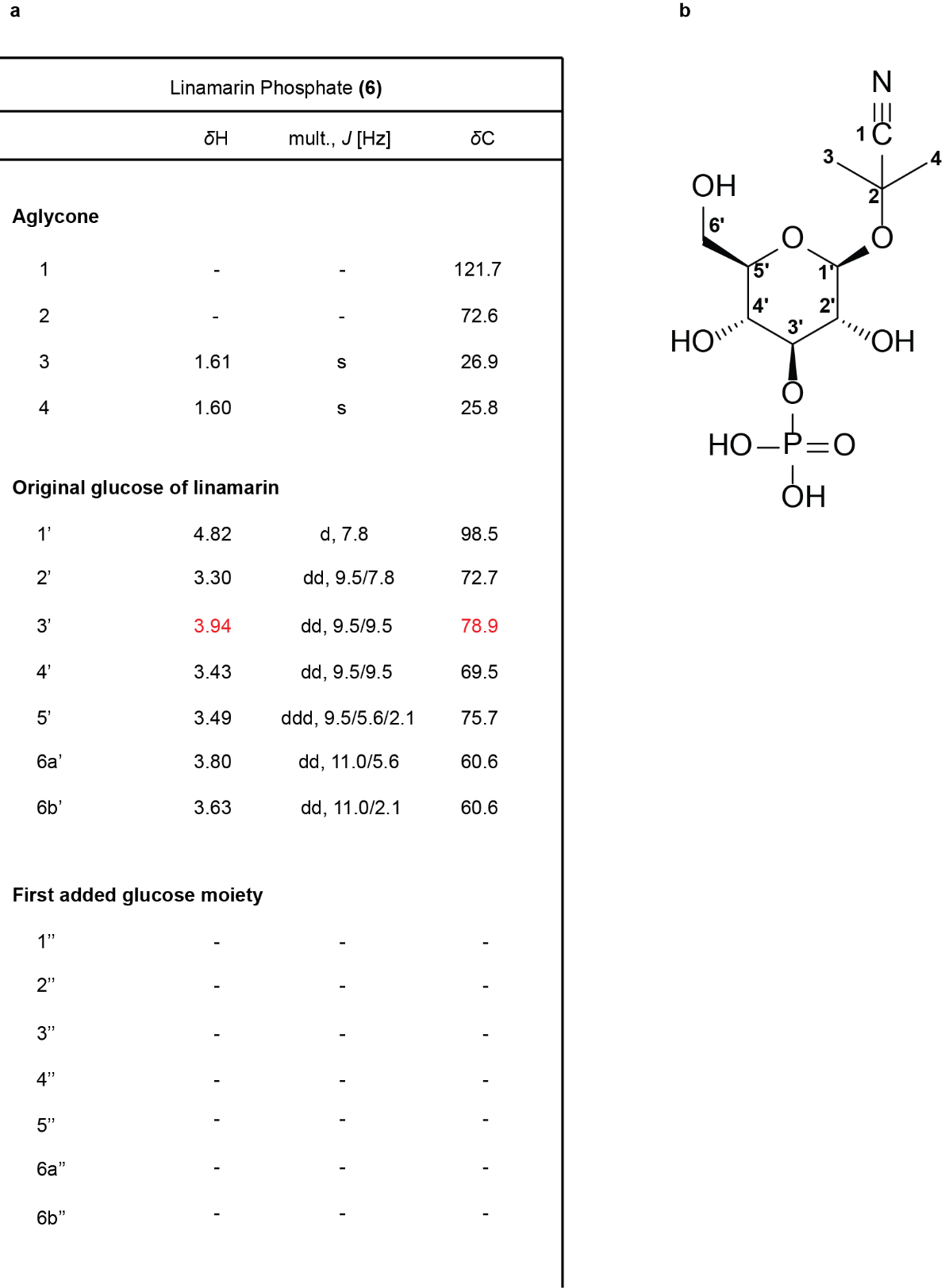


**Figure SN1-5:** (**a**) ^1^H and ^13^C NMR shifts and coupling constants for phopshorylated linamarin (**6**) and its structure (**b**) with numbering.


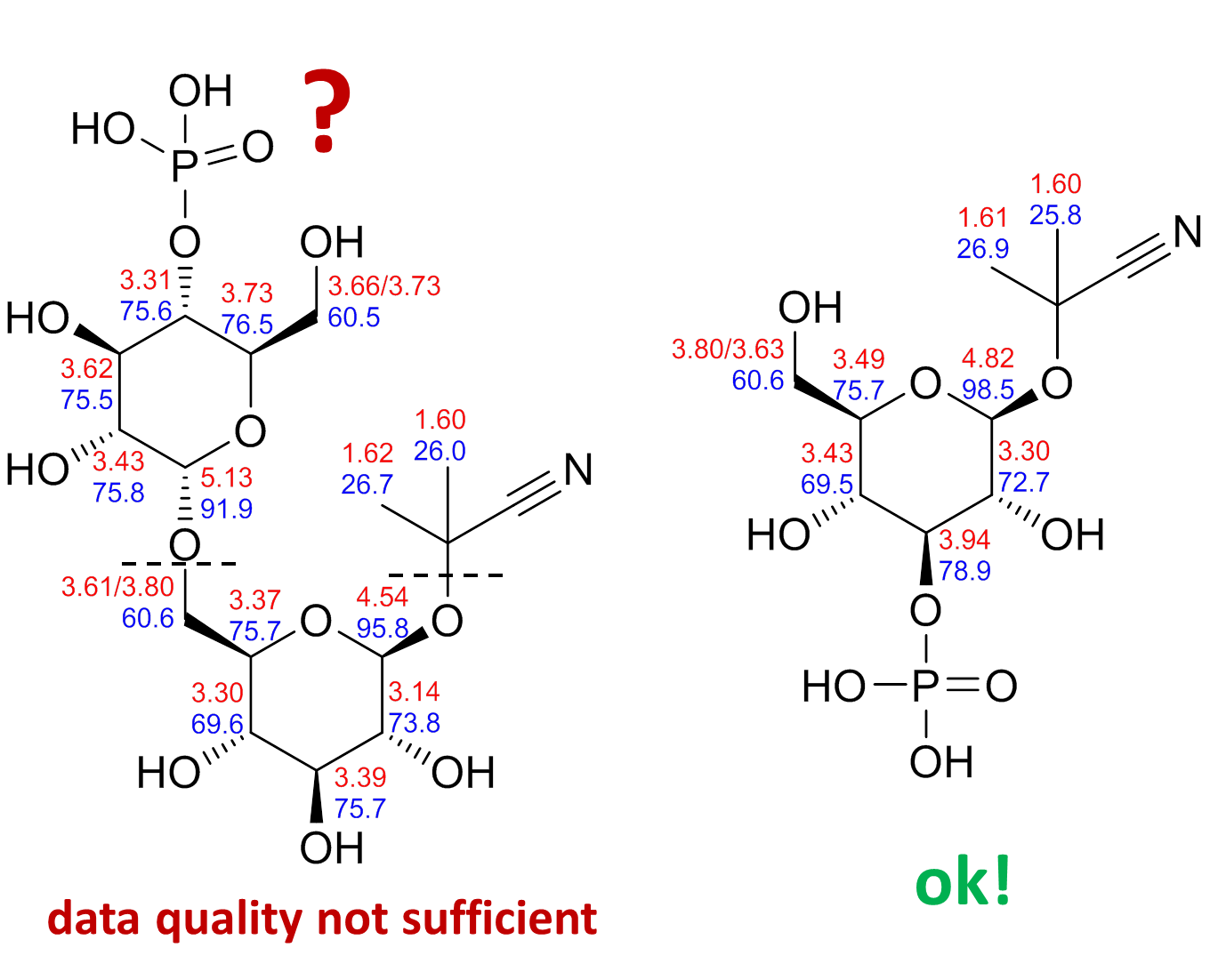


**Figure SN1-6:** Structure and chemical shifts of linamarin-3’-*O*-phosphate (**6**). ^1^H chemical shifts in red, ^13^C chemical shift in blue.


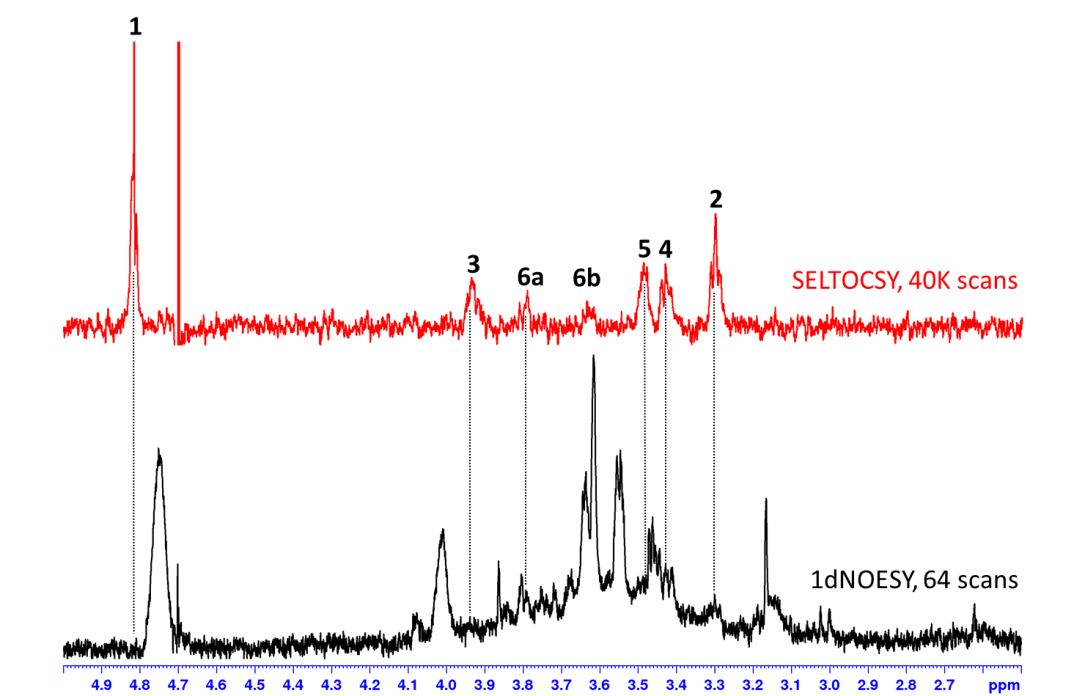


**Figure SN1-7:** Details of the 1dNOESY (black trace) and a SELTOCSY spectrum of linamarin-3’-*O*-phosphate (700 MHz, in D_2_O). Numbers indicate the position in the glucose part of the molecule. The transmitter frequency for the SELTOCSY experiments (position one of the glucose part) was extracted from an HSQC experiment (figure SN1-9).


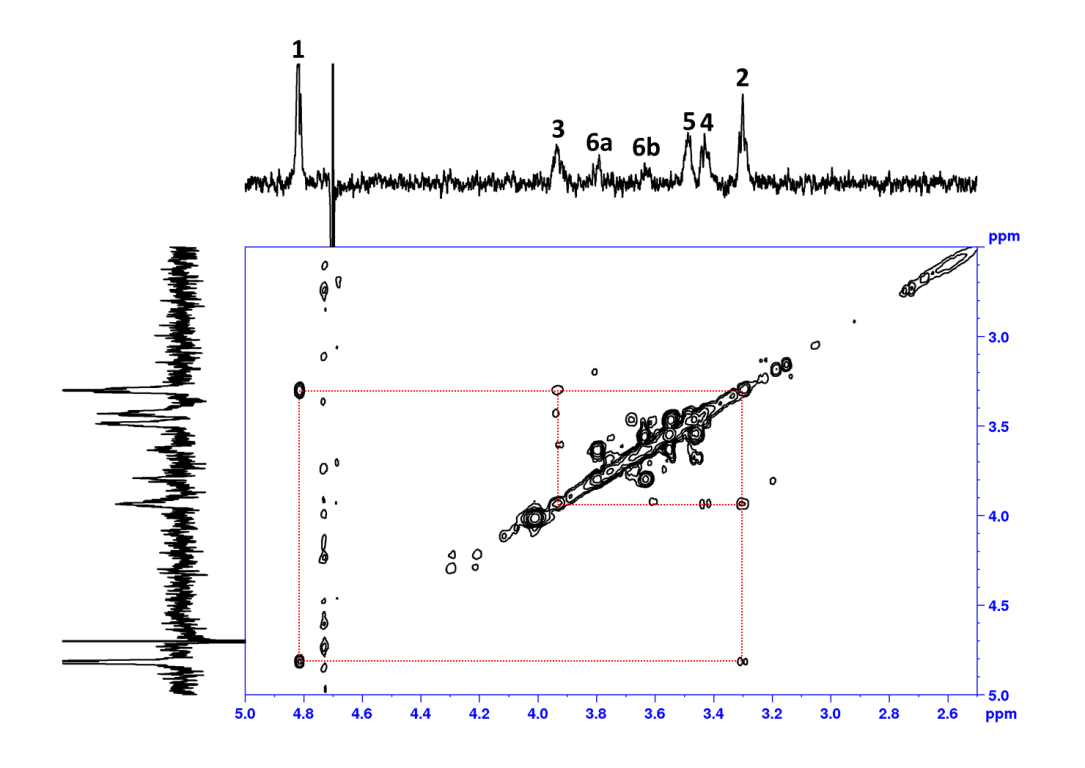


**Figure SN1-6:** Detail of the DQFCOSY of linamarin-3’-*O*-phosphate (700 MHz, magnitude mode, in D_2_O). The SELTOCSY from Fig. SN1-7 served as F1- and F2-projection


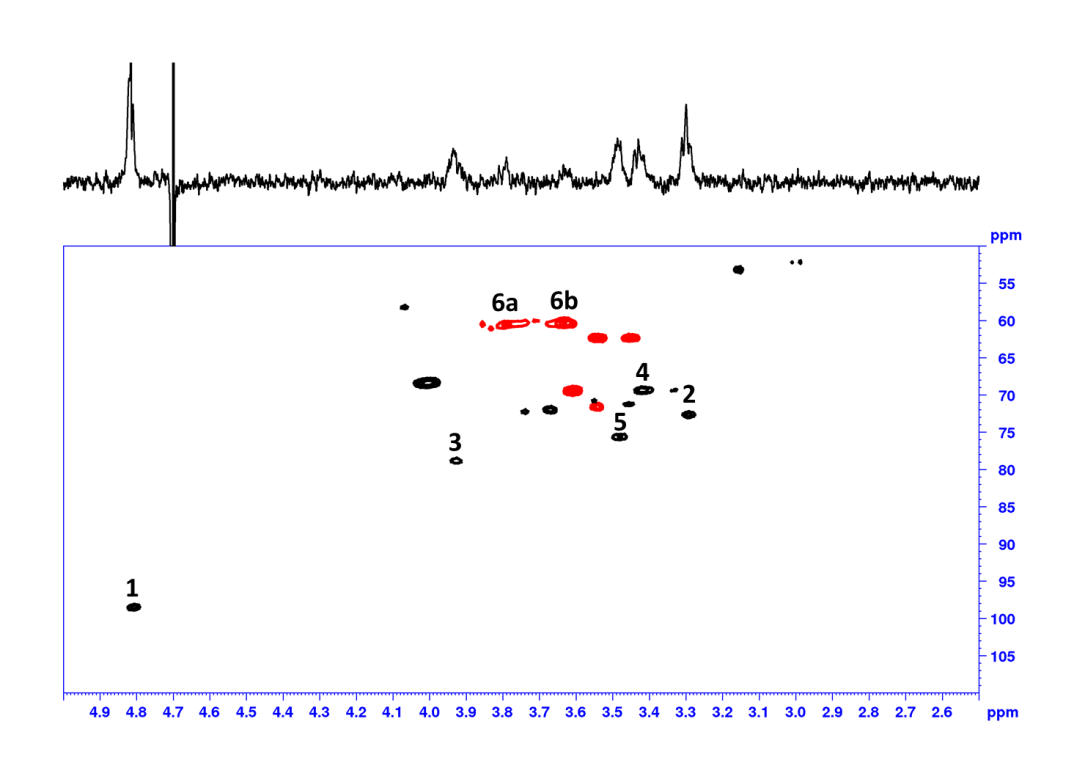


**Figure SN1-9:** Detail of the ^1^H-^13^C HSQC spectrum of linamarin-3’-*O*-phosphate. The SELTOCSY from Fig. SN1-7 served as F2-projection.


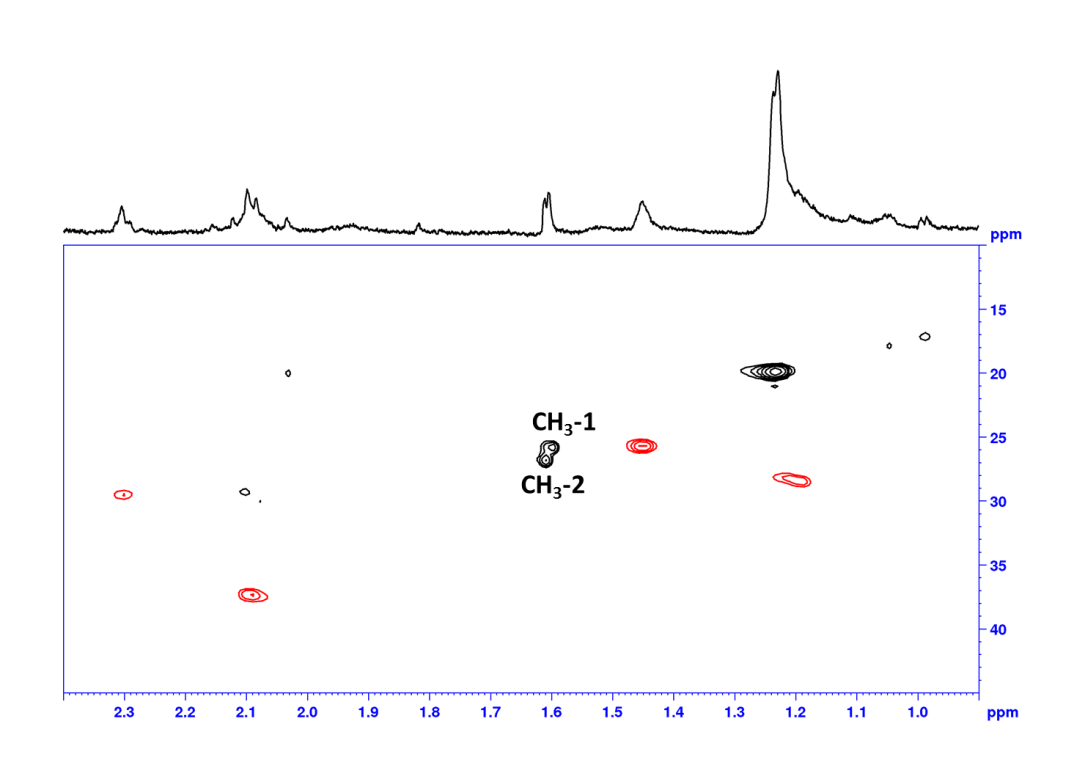


**Figure SN1-10:** Detail of the ^1^H-^13^C HSQC spectrum of linamarin-3’-*O*-phosphate. The 1dNOESY from Fig. SN1-7 served as F2-projection.


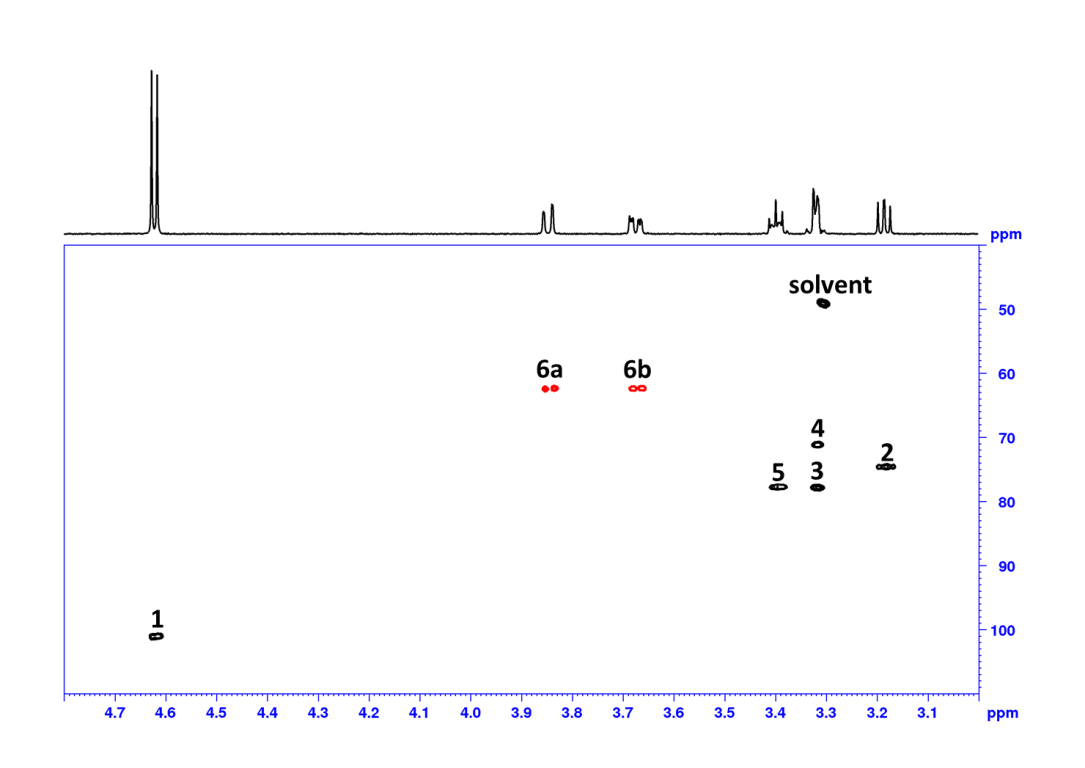


**Figure SN1-11:** Detail of the ^1^H-^13^C HSQC spectrum of linamarin (in MeOH-*d_3_*). A SELTOCSY (transmitter on pos.1 of the glucose part) was used as F2-projection. The spectrum illustrates the chemical shifts of position 3 of the glucose part without phosphorylation.
